# Novel carboxylate-based glycolipids: TLR4 antagonism, MD-2 binding and self-assembly properties

**DOI:** 10.1038/s41598-018-37421-w

**Published:** 2019-01-29

**Authors:** Florent Cochet, Fabio A. Facchini, Lenny Zaffaroni, Jean-Marc Billod, Helena Coelho, Aurora Holgado, Harald Braun, Rudi Beyaert, Roman Jerala, Jesus Jimenez-Barbero, Sonsoles Martin-Santamaria, Francesco Peri

**Affiliations:** 1 Department of Biotechnology and Biosciences, University of Milano-Bicocca, Piazza della Scienza, 2, 20126 Milano, Italy; 2 Department of Structural and Chemical Biology, Centro de Investigaciones Biologicas, CIB-CSIC, Ramiro de Maeztu, 9, 28040 Madrid, Spain; 3 Molecular Recognition & Host-Pathogen Interactions Programme, CIC bioGUNE, Bizkaia Technology Park, Building 801 A, 48170 Derio, Spain; 4 UCIBIO, REQUIMTE, Departamento de Quimica, Faculdade de Ciencias e Tecnologia, Universidade Nova de Lisboa, 2829-516 Caparica, Portugal; 5 Department of Organic Chemistry II, Faculty of Science & Technology, University of the Basque Country, 48940 Leioa, Bizkaia Spain; 6 Unit for Molecular Signal Transduction in Inflammation VIB-UGent Center for Inflammation Research, VIB Technologiepark 927, 9052 Zwijnaarde, Ghent Belgium; 7 Department of Biomedical Molecular Biology, Ghent University Technologiepark 927, 9052 Zwijnaarde, Ghent Belgium; 8 Department of Biotechnology, National Institute of Chemistry, Hajdrihova 19, 1000 Ljubljana, Slovenia; 9 Molecular Recognition & Host−Pathogen Interactions Programme, CIC bioGUNE, Bizkaia Technology Park, Building 801 A, 48170 Derio, Spain; 10 Department of Organic Chemistry II, Faculty of Science & Technology, University of the Basque Country, 48940 Leioa, Bizkaia Spain; 11 Ikerbasque, Basque Foundation for Science, Maria Diaz de Haro 13, 48009 Bilbao, Spain

## Abstract

New monosaccharide-based lipid A analogues were rationally designed through MD-2 docking studies. A panel of compounds with two carboxylate groups as phosphates bioisosteres, was synthesized with the same glucosamine-bis-succinyl core linked to different unsaturated and saturated fatty acid chains. The binding of the synthetic compounds to purified, functional recombinant human MD-2 was studied by four independent methods. All compounds bound to MD-2 with similar affinities and inhibited in a concentration-dependent manner the LPS-stimulated TLR4 signaling in human and murine cells, while being inactive as TLR4 agonists when provided alone. A compound of the panel was tested *in vivo* and was not able to inhibit the production of proinflammatory cytokines in animals. This lack of activity is probably due to strong binding to serum albumin, as suggested by cell experiments in the presence of the serum. The interesting self-assembly property in solution of this type of compounds was investigated by computational methods and microscopy, and formation of large vesicles was observed by cryo-TEM microscopy.

## Introduction

The Toll-like Receptor 4 (TLR4) is the mammalian receptor responsible for the Gram-negative bacterial endotoxin recognition (lipopolysaccharide, LPS and lipooligosaccharide, LOS). TLR4 is mainly expressed on the cells surface of innate immune^1^ and epithelial cells^2^, allowing them to sense minute amounts of LPS released by the Gram-negative bacteria. An ordered series of interactions among the lipophilic portion of LPS and LBP^3,4^, CD14^5,6^ and MD-2^7–10^ co-receptors allows the formation of the activated membrane heterodimeric complex (LPS/MD-2/TLR4)_2_^11^ that triggers an intracellular signal^12^ inducing the production of pro-inflammatory cytokines and chemokines^13,14^. TLR4-mediated cytokine production is an essential mechanism by which the host organism responds to infections, however, excessive stimulation of TLR4 by pathogen-associated molecular patterns (PAMPs) can cause uncontrolled cytokine production leading to serious life-threatening syndromes such as acute sepsis and septic shock^15^. Recently, TLR4 activation by endogenous factors (DAMPs) has been associated to several inflammatory disorders and auto-immune diseases affecting a variety of organs and body functions^16–18^. In this context, the development of hit or lead compounds that are able to modulate TLR4 signaling is attracting increasing interest for a wide range of possible therapeutic settings^19^. TLR4 antagonists of synthetic or natural origin can block TLR4 signaling by interacting with the natural TLR4-bound co-receptor MD-2^20^, thus competing with the natural ligand LPS. Other TLR4 inhibition mechanisms are based on preventing LPS-induced receptor co-localization and dimerization (MGCs)^21^, on interfering with cytosolic adaptor protein recruitment (TAK242)^22^, or on the direct binding with other co-receptors such as the CD14 co-receptor^23–26^.

The type of modulation (agonism or antagonism) and the potency of TLR4 modulation by lipid A (phospholipidic part of LPS recognized by TLR4) and lipid A analogues not only depends on the interaction with CD14 and MD-2 receptors, but also on the aggregation state in solution of such amphiphilic molecules. The size and 3D shape of aggregates directly influences early stages of ligand recognition, namely the interaction with LBP and CD14 receptors^27^. Large lamellar or spherical aggregates have been associated respectively to the antagonist and agonist behavior of the lipid A variants^26,28–30^.

**Figure 1:**
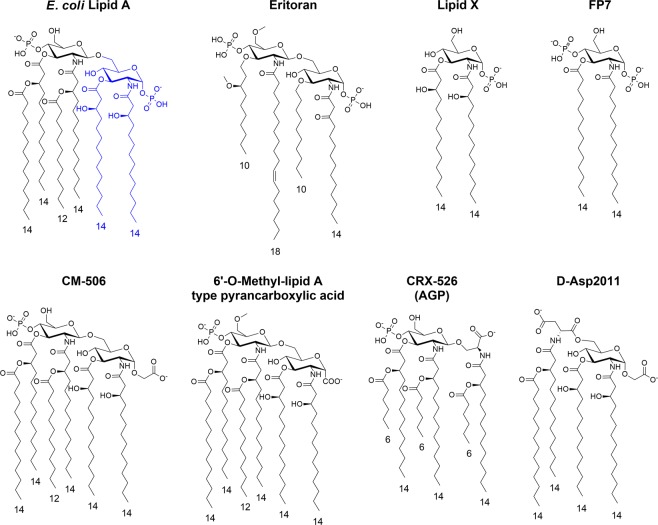
Chemical structures. Natural *E*. *coli* lipid A, lipid X, synthetic antagonist E5564 (Eritoran) and monosaccharide FP7. Synthetic Lipid A mimics with carboxylic acids replacing phosphates, active as TLR4 modulators.

Compound E5564 (Eritoran, Fig. 1)^31^ is one of the most potent TLR4 inhibitors so far. The activity of this molecule is associated to its capacity to mimic the lipid A moiety, thus competing with LPS for MD-2 binding. Eritoran consists of a glucosamine disaccharide with two phosphate groups, in C1 and C4′ positions, and four lipophilic chains. Other TLR4 antagonists have glycolipid structures, as in the case of Gifu monosaccharides^32^, Lipid X and compound FP7 (Fig. 1)^25,33^ or have a chemical structure unrelated to lipid A^34^. Eritoran and FP7 are the only lipid A mimetics whose direct binding to MD-2 and the competition with natural MD-2 ligands LPS and LOS have been reported^20,26,35,36^.

With the aim of obtaining lipid A mimetics with drug-like features, including increased metabolic stability, the anionic phosphate group has been replaced by the bioisosteric carboxylate group (Fig. 1)^37–43^ and different Lipid A analogues have been reported presenting different acylation patterns together with a carboxymethyl^37–40^ or a carboxyl group linked to the anomeric carbon^41^. In the case of aminoalkyl glucosaminide-4-phosphates (AGPs, or Corixa compounds, CRX)^44^, the whole reducing sugar and phosphate have also been replaced by an acylated diethanolamine bound to a phosphate or a carboxylic acid^42,43^.

AGPs act as TLR4 agonists or antagonists depending on the acylation patterns and the fatty acids chains lengths, and the variants with agonist properties (CRX 526, Fig. 1) were subsequently developed as vaccine adjuvants^45^. The TLR4 agonistic/antagonist activity of AGPs is a good indication that the bioisosteric substitution of the phosphate group in C1 by a carboxylic acid, preserves the ability to bind to MD-2 and triggers or inhibits TLR4 dimerization. While some of carboxylic acid synthetic variants of lipid A have been characterized for their immunomodulating activity in cells and in animal models, no data are available on the characterization of their direct binding to MD-2.

**Figure 2:**
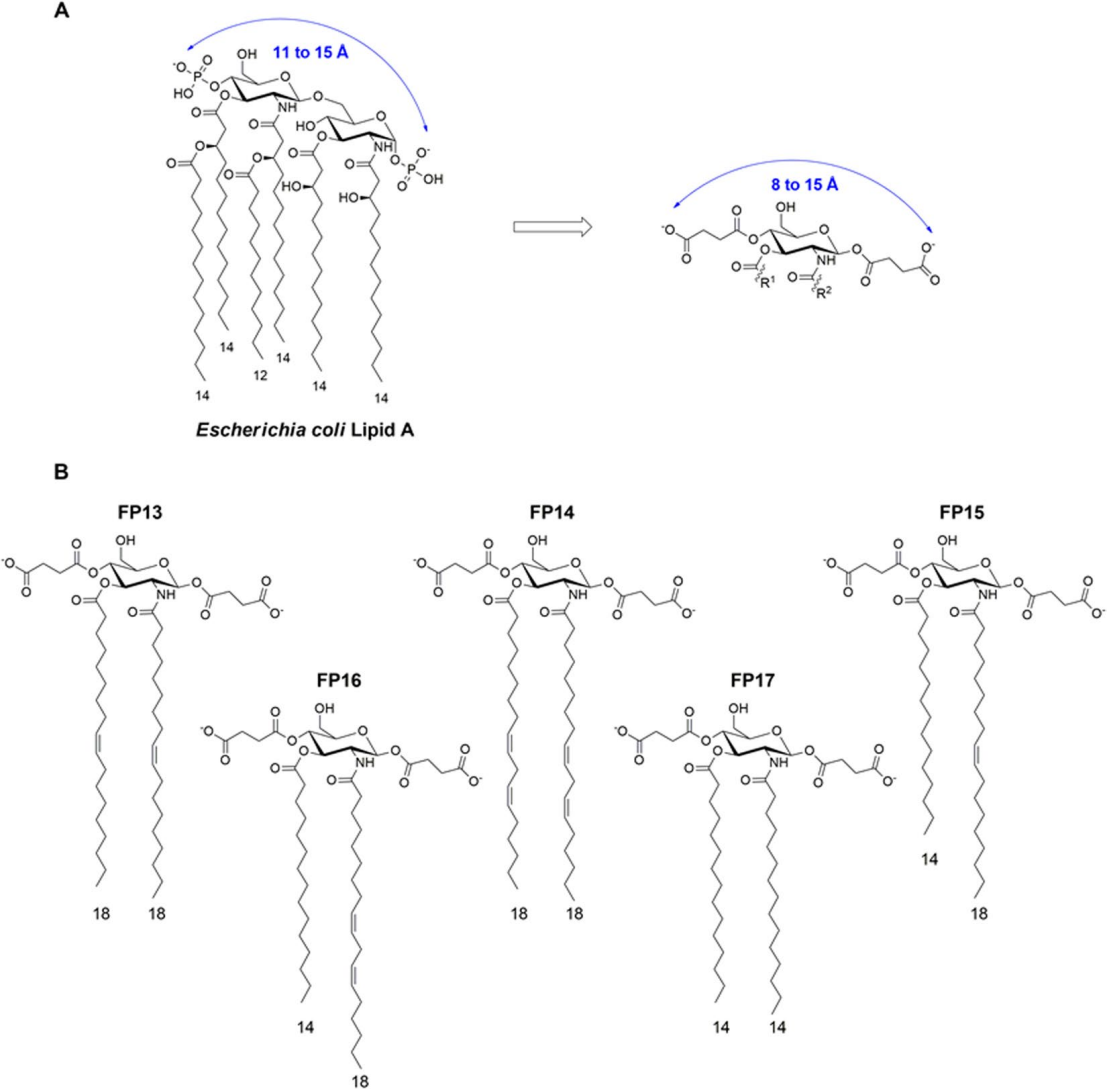
(**A**) Monosaccharide FP13-17 are lipid A mimetics. (**B**) FP13 contains two oleic acid chains (C18, cis-9), FP14 two linoleic acid chains (C18, cis, cis-9,12), FP15 myristic acid at C3 (C14) and oleic acid at C2, FP16 myristic acid at C3 and linoleic acid at C2 and FP17 two myristic acid chains.

We report here a small series of monosaccharide-based lipid A mimetics resembling FP7, with carboxyl moieties mimicking phosphates and with unsaturated fatty acid chains. We tested the capacity of this variety of molecules to interact with the MD-2 co-receptor in multiple binding assays and we evaluated their biological activity on different cellular models. Compounds FP13- FP17 (Fig. 2) have been rationally designed as lipid A mimetics where the phosphates have been replaced by succinate moieties, while the distance between the two carboxylate groups has been kept similar to that of the two phosphates groups in lipid A (distance of 11 to 15 Å for *E*. *coli* Lipid A and 8 to 15 Å for FP13-17)^37–39,41–43^. Moreover, unsaturated chains (also present in Eritoran) have also been inserted to enhance the binding into the MD-2 hydrophobic pocket^46^.

All compounds are based on a glucosamine scaffold, and present two units of succinic acid condensed to the hydroxyl groups at C1 and C4. As the TLR4 antagonist Eritoran presents an unsaturated chain (C18, cis-11) that has the role to increase the binding affinity for the MD-2 hydrophobic pocket^47^, we aimed to investigate the effect of unsaturated chains linked to glucosamine C2 and C3. FP13 contains two oleic chains (C18, cis-9), FP14 two linoleic chains (C18, cis, cis-9,12), FP15 myristic at C3 (C14) and oleic at C2, FP16 myristic at C3 and linoleic at C2 and FP17 two myristic chains (Fig. 2).

## Results

### Computational studies of the binding of FP13-17 to TLR4/MD-2

**Figure 3:**
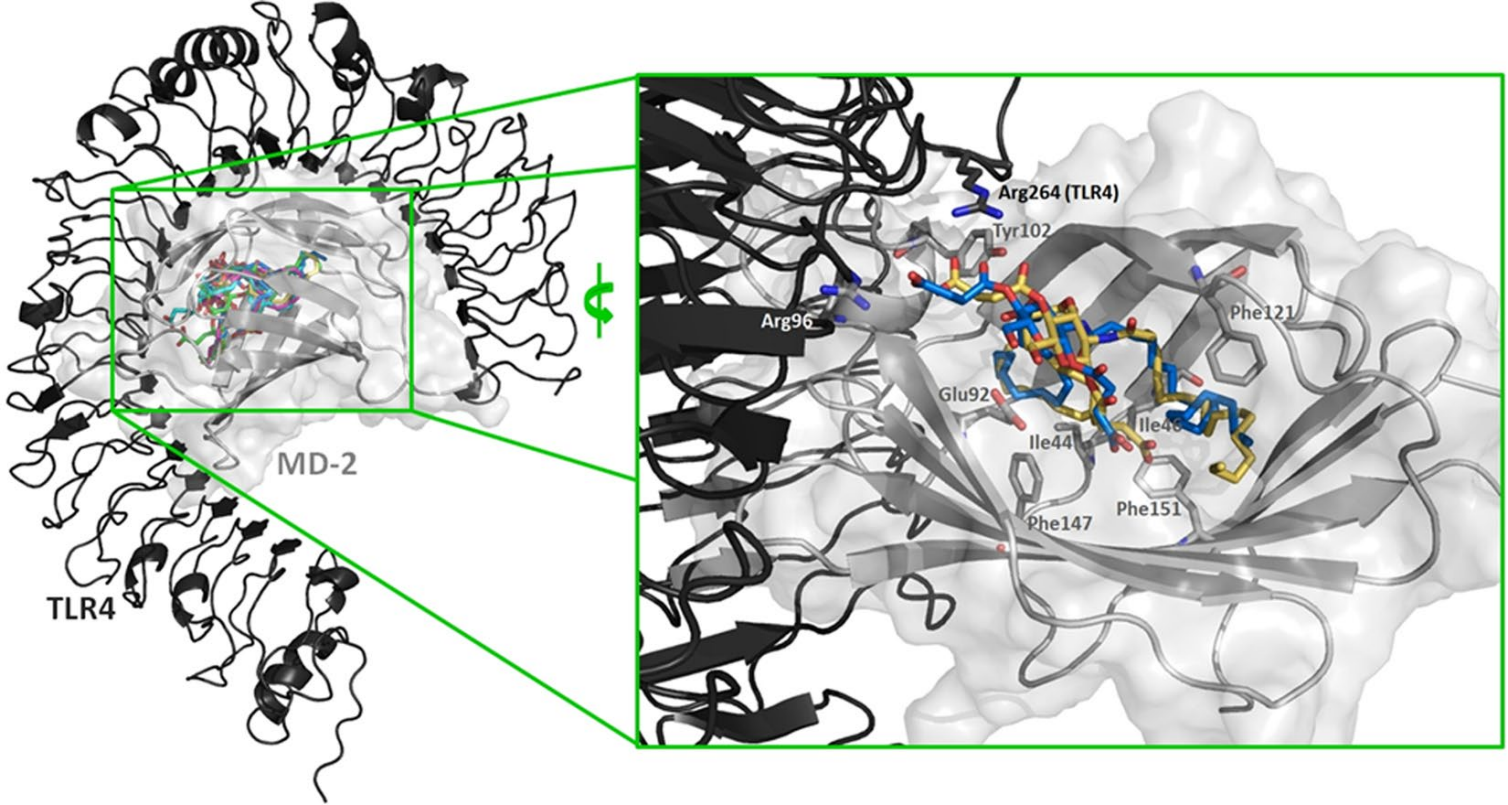
Best AutoDock Vina docked poses of FP13-17 within the TLR4/MD-2 complex. TLR4 and MD-2 are respectively represented in black and grey cartoon. Ligands FP13 to FP17 are in yellow, cyan, pink, blue and green sticks, respectively (left: general view; right: detailed view showing selected interacting residues, only FP13 in yellow and FP16 in blue are shown).

Designed monosaccharides FP13-17 were computationally studied to predict their TLR4/MD-2 binding properties. To explore their possible binding modes, the compounds were computationally docked in a 3D model of the TLR4/MD-2 complex in antagonist conformation previously reported by us^48^, by means of the Vina docking program^49^. Compounds FP13-17 were predicted to bind inside the MD-2 hydrophobic pocket (Fig. 3) with favorable predicted binding scores (ranging from −8.2 to −6.7 kcal/mol for the 20 best predicted poses). Two different binding orientations were identified: one similar to that for *E*. *coli* lipid A in PDB ID 3FXI, and a second one similar to that for antagonist lipid IVa in PDB ID 2E59 (rotated by 180 degrees along the lipid chains axis compared with agonist *E*. *coli* LPS in PDB ID 3FXI, Supp. Inf. Fig. S1). In all case, the fatty acid chains were inserted inside the MD-2 pocket establishing interactions with the aliphatic and aromatic residues. In particular, Phe151 was found to interact with the unsaturated moiety of oleic and linoleic acids in compounds FP13-17, while other hydrophobic interactions were found with side chains from Phe121, Phe147, Ile44 and Ile46 residues (Fig. 3). Regarding the polar moieties of FP13-17 (pyranose ring of the sugar and esters groups), they were participating in a variety of polar interactions with the polar residues at the MD-2 rim, such as Tyr102 and Glu92. The carboxylate groups were predicted to establish ionic interactions with Arg96 and Arg264 (TLR4) in most of the docked poses for all the compounds (Fig. 3).

Selected docked binding poses obtained with Vina were submitted to re-docking with AutoDock^50^, another well-known docking program with a different scoring function. Fittingly, the best poses obtained for each ligand were in good agreement with those obtained through Vina, which supported the appropriateness of the employed approach. Predicted scores ranged from −7.38 to −4.53 kcal/mol across all the ligands with a score for the best predicted poses ranging from −7.38 to −4.69 kcal/mol. No major score or binding differences were observed among the set of ligands suggesting similar binding properties.

To study in detail the binding interactions of each ligand to the TLR4/MD-2 system, we performed molecular dynamics (MD) simulations of the complexes of TLR4/MD-2 with the best docked pose for each ligand. The dynamics of the ligands and of key residues of MD-2 were analyzed. MD-2 is stable over the trajectory as shown by the RMSD and RMS fluctuation per residues analysis (Supp. Inf. Fig. S2). Polar interactions are maintained along the simulation and residue Phe126 does not undergo major conformational change (Supp. Inf. Fig. S3), indicating that all the ligands are able to retain the antagonist conformation of MD-2, previously associated with antagonist properties^11^. Interestingly, the starting docked poses relocate, after 50 ns of MD simulation, from a slightly deep position inside the MD-2 rim towards a binding mode closer to that for lipid IVa (depicted for FP13 in Supp. Inf. Fig. S4). These similar modes of TLR4/MD-2 binding were in agreement with a similar antagonist activity (see below).

The solubility of the ligands, which is a critical property for cellular assays, was also analyzed by calculating the logP parameter (Supp. Inf. Fig. S5). The results point toward relatively high logP values. However, they are in the range of a previously reported family of FP7 analogues^51^. Despite the high calculated logP it was however possible to dissolve all compounds in aqueous buffers and test them on cells and animals.

### MD-2 binding properties of synthetic compounds

Compounds FP13-17 were synthesized according to multistep synthesis (Fig. S6, Supp. Info). and were tested for their capacity to bind to purified human MD-2 (hMD-2) Four different techniques were used: two ELISA-type plate-based assays with immobilized protein, a fluorescence displacement assay, and surface plasmon resonance (SPR)^52,53^.

**Figure 4:**
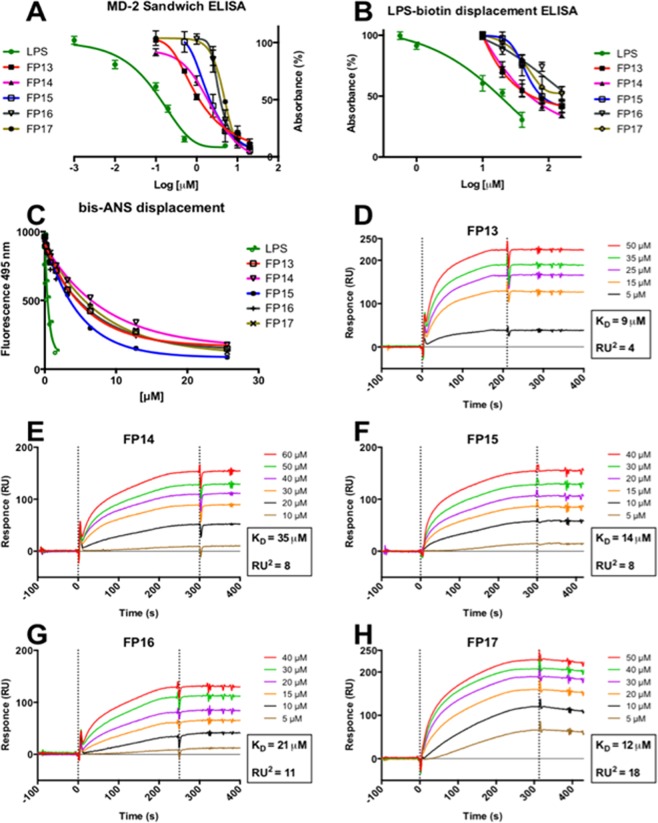
Binding studies on purified hMD-2 receptor. (**A**) FP13-17 prevent anti-human hMD-2 monoclonal antibody binding in a dose-dependent manner; (**B**) FP13-17 compete with biotin-LPS for hMD-2 binding; (**C**) FP13-17 dose-dependently inhibit the binding of bis-ANS to hMD-2;(**D**)-(**H**). SPR analysis show direct interaction between FP13-17 and hMD-2; K_D_ values are reported.

**Figure 5:**
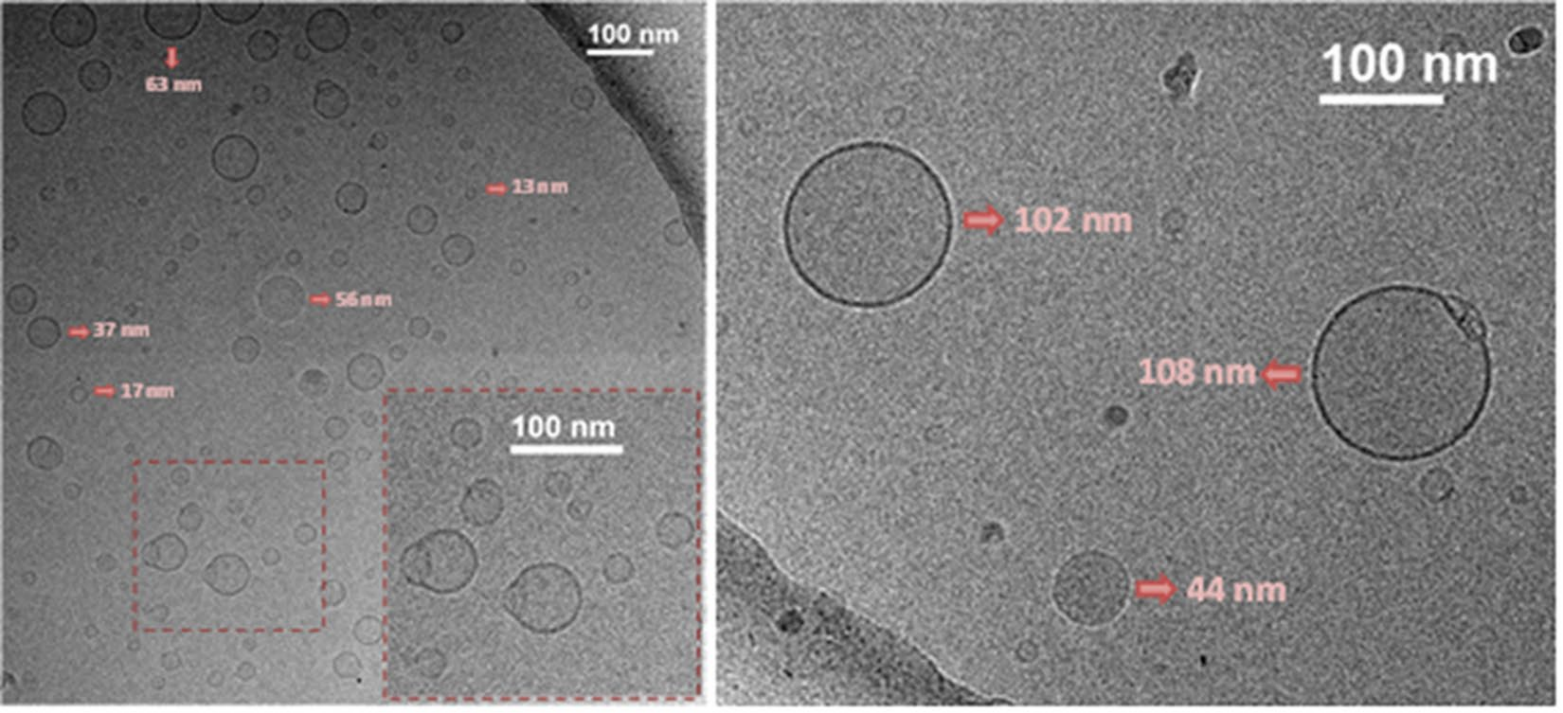
Cryogenic Transmission Electron Microscopy (Cryo-TEM) of FP15 (7 mg/mL) nominal magnification of 40,000× (0.26 nm/pixel).

Direct binding of LPS and synthetic molecules FP13-17 to hMD-2 was determined by using a monoclonal antibody that binds to free hMD-2 but not to LPS bound to the hMD-2 binding site^54^. Monoclonal mouse anti-hMD-2 (9B4) antibody specifically binds to an epitope close to the rim of the hMD-2 pocket, available for the binding only when the hMD-2 pocket is empty. This assay detected a decrease in binding to hMD-2 in the presence of LPS (Fig. 5A), similar to what has previously been reported^55^. A dose-dependent inhibition of antibody/MD-2 interaction was observed when adding molecules FP13-17, with a 85−95% decrease in binding obtained at concentrations of molecules FP13-17 of 20 μM (Fig. 4A). This indicates that the synthetic molecules bind the cavity of MD-2.

The ability of synthetic molecules FP13-17 to displace LPS from the hMD-2 pocket was assessed by ELISA. FP13-17 molecules were added at increasing concentrations to hMD-2 that was previously incubated with biotinylated LPS. FP13-17 molecules were able to displace biotin-LPS from the hMD-2 pocket in a similar dose-dependent manner, with the highest displacement of 40−55% obtained at a concentration of 160 μM (Fig. 4B). As a control, LPS at a concentration of 40 μM gave the highest displacement of biotin-LPS of 70% (Fig. 4B).

It has been previously shown that the fluorescent probe 1,1′-Bis(anilino)−4,4′-bis (naphthalene)−8,8′ disulfonate (bis-ANS) binds to hMD-2 and it is displaced by LPS^53^. bis-ANS binds the same hMD-2 binding pocket that accommodates the fatty acid chains of lipid A and of other lipid A-like ligands, so that TLR4 modulators interacting with MD-2 in a lipid A-like manner, compete with bis-ANS and displace it from hMD-2. Synthetic compounds FP13-17 caused a similar concentration-dependent decrease of bis-ANS fluorescence, indicating competitive binding of molecules FP13-17 to hMD-2 (Fig. 4C).

Results of SPR data experiments based on binding of compounds to the immobilized hMD-2, showed direct interaction of the hMD-2 receptor with LPS (control, Supp. Inf. Fig. S7) and with the tested synthetic compounds FP13-17. K_D_ values derived from sensorgram analysis were 9, 35, 14, 21 and 12 μM for FP13, FP14, FP15, FP16 and FP17, respectively (Fig. 4D–H). SPR experimental curve optimal fitting was obtained by assuming 1:1 ligand/MD-2 binding stoichiometry.

Together, the results obtained from these *in vitro* cell-free studies clearly indicate that compounds FP13-17 directly bind to hMD-2.

### Cryogenic Transmission Electron Microscopy (Cryo-TEM)

We have recently described the aggregation behavior of FP7 glycolipid, a related molecule to those described herein^51^. In particular, the analysis of the obtained NMR and TEM data (cryogenic and negative staining) permitted to show that FP7 forms micelle structures^36^. Indeed, previous investigations had demonstrated that FP7 has a CMC of about 9 µM^56^. Thus, the self-assembly in solution of one member of the series, namely compound FP15, was studied by Cryogenic Transmission Electron Microscopy (Cryo-TEM) (Fig. 5).

The detailed inspection of the cryo-TEM data shows that FP15 mainly forms spherical and homogeneous small unilamellar vesicles (SUVs), although with rather different size distributions, from 10 to 110 nm. Moreover, it was possible to detect the presence of fusion events (as highlighted in the zoomed picture in Fig. 5, dark red squares) as well as the existence of open bilayers. The use of vitrified samples allowed the trapping of the potentially unstable structures associated with the formed intermediates in the solubilization process of the vesicles.

These experimental data show that FP15 is able to form vesicles/liposomes displaying a bilayer.

### Molecular dynamics (MD) simulations of FP15 self-assembly

**Figure 6:**
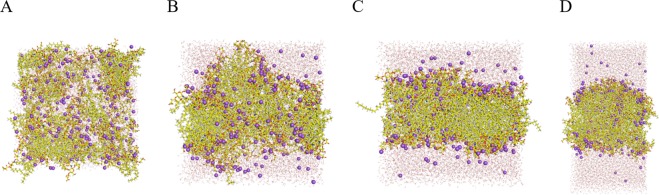
Representation of the evolution of the FP15-water mixture over time, t = 0 ns (**A**), t = 50 ns (**B**), t = 200 ns (**C**) and t = 1000 ns (**D**). FP15 in represented in CPK colored sticks, ion Na^+^ in violet spheres and the water molecules in thin grey and red lines.

As FP15 was experimentally shown to spontaneously form SUVs, observed through Cryo-TEM experiments, we were eager to understand this phenomenon at an atomistic level by performing MD simulations of FP15 in water (see Experimental Section for details). A thorough explanation and discussion about all the self-assembly simulations performed is given in Supp. Inf., the following analysis is based on one unique selected long simulation of 1 µs in total. Starting from a random mixture of FP15 molecules and explicit water molecules, the system was observed to self-organize into a bilayer system, reaching a stable regime from 700 ns of simulation (according to the area per molecule graph, Supp. Inf. Fig. S8, left). For this reason, only the last 300 ns were considered for plotting electron density profile (Supp. Inf. Fig. S8, right) to estimate the thickness of the FP15 layer between 40 and 70 Å. It can be grasped from Fig. 6 that the membrane is slightly curved resulting in an electron density profile with soft delimitation between the water and the FP15 phases preventing us form precisely assessing the thickness. The graph also shows that water is present at all latitude of the membrane with a minimum at the center indicating that the vesicles might be highly permeable to water.

In each layer, FP15 is regrouped in small cluster surrounded by water accessible pores. The smallest, and what looks like the most stable cluster, is a face-to-face head group packaging assembly of two FP15 units, stabilized through hydrogen bonds and interactions between carboxylate anion from the succinate groups through Na^+^ ionic interactions. Two other assemblies of four FP15 are observed, one in which the saccharide rings are packed in parallel, being stabilized by hydrogen bonds and ionic interactions, the other one is an in-circle assembly of FP15, around a spot rich in Na^+^ ions, driven exclusively by ionic interactions between succinate groups and Na^+^ ions (Supp. Inf. Fig. S9). Additionally, we can observe that the unsaturation in the acyl chains induces a sharp turn at the center of the membrane, preventing the different units of FP15 to form highly organized bilayers.

### Inhibition of LPS-stimulated human TLR4 activation in HEK293-Blue cells

**Figure 7:**
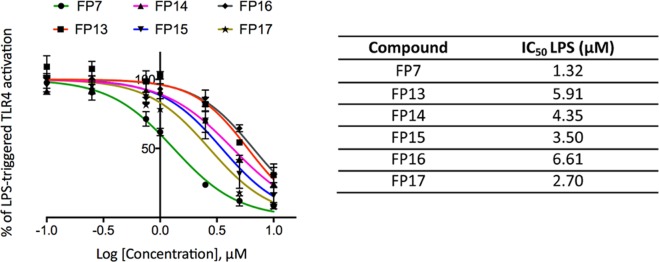
Dose-dependent inhibition of LPS-triggered TLR4 pathway activation in HEK293-Blue hTLR4 cells. Cells were pre-incubated with increasing concentrations (0.1 to 10 µM) of compounds FP13-17 in serum-free DMEM medium and stimulated with LPS (100 ng/mL) after 15 min. SEAP levels in cell culture media were quantified after 16 hours as indicator of TLR4 activation. Data were normalized to stimulation with LPS alone and fitted to a sigmoidal 4 parameter logistic equation to obtain dose-effect curves (left panel). IC_50_ values for FP13-17 are reported in the right panel. points represent the mean of percentage ± SEM of at least 3 independent experiments.

Synthetic monosaccharides were screened for their capacity to activate or inhibit LPS-stimulated TLR4 activation in HEK293-Blue hTLR4 cells. HEK293-Blue hTLR4 cells are HEK cells transfected in order to express the human receptors involved in LPS detection process (TLR4, MD-2 and membrane-bound CD14) and an inducible SEAP (secreted embryonic alkaline phosphatase) reporter gene placed under the control of NF-kB and AP-1-transcription factors. Activation of the MyD88-dependent TLR4 pathway promotes the production and secretion of SEAP in the cell culture media, which can be easily quantified. In order to evaluate the capacity of FP13-17 to trigger TLR4 activation, cells were treated with increasing concentrations of the compounds (0.1 to 10 µM) and incubated for 16 hours. On the other hand, to evaluate the antagonist activity, cells were pre-incubated with compounds FP13-17 for 15 min. and stimulated with LPS for 16 hours. The results obtained revealed that none of the molecules induced TLR4 signaling up to a concentration of 10 μM, thus indicating lack of agonist activity (Data not shown). Conversely, FP13-17 did inhibit, in a dose-dependent manner, the LPS-induced TLR4 signaling (Fig. 7). All molecules showed similar antagonist potency (IC_50_ values is in the range 2.7 to 6.6 µM, also in the same order of magnitude than that of IC_50_ of FP7, range, 0.46-3.42 µM)^51,56^. An MTT assay revealed that the compounds did not affect cell viability at concentrations up to 10 μM (Supp. Inf. Fig. S10).

### Inhibition of LPS signaling in murine macrophages

**Figure 8:**
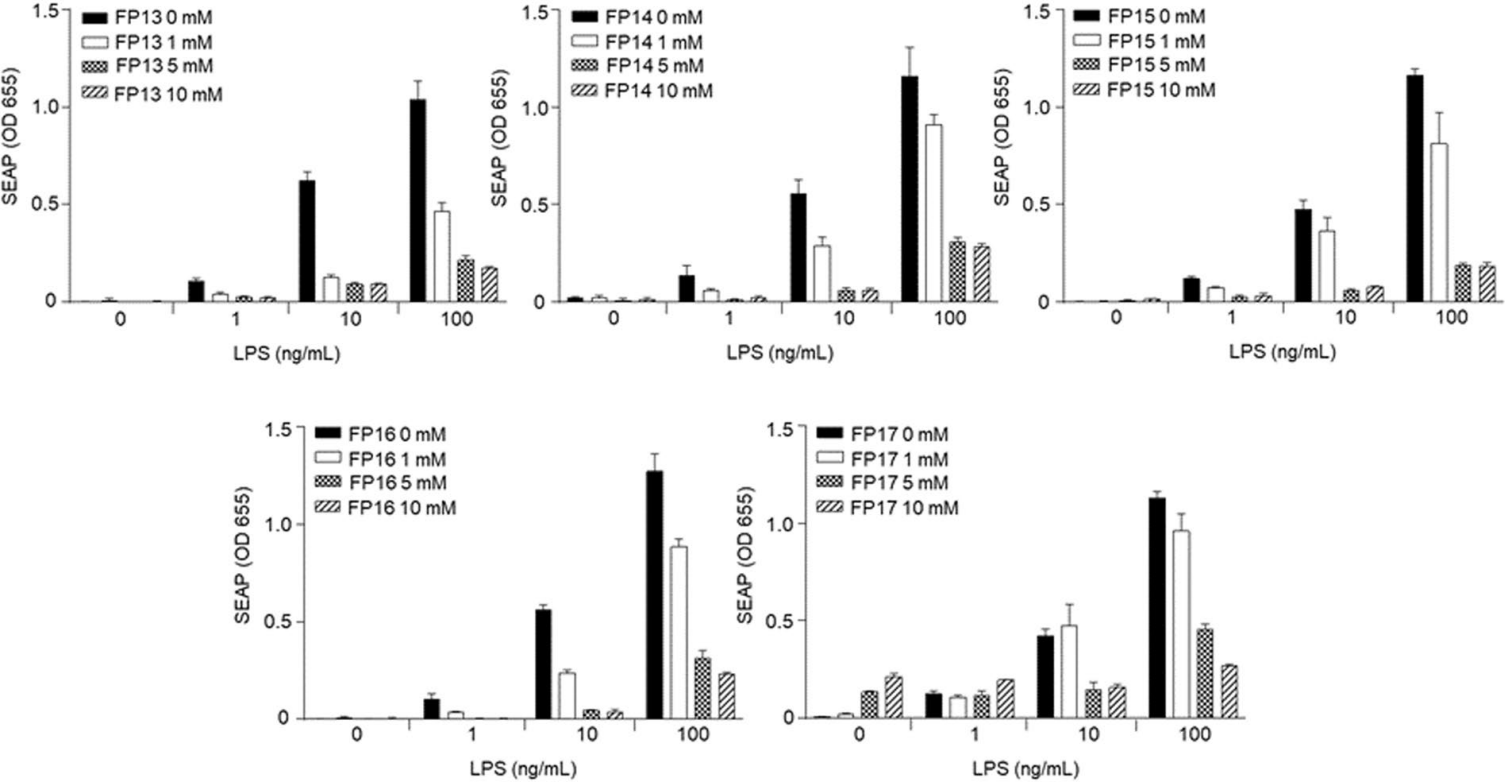
Inhibition of LPS-induced TLR4 signaling in RAW 264.7-Blue cells. Cells were pre-incubated with the indicated concentrations of monosaccharides FP13-17 and stimulated with 1, 10 or 100 ng/mL *E*. *coli* O111:B4 LPS 30 min. later in serum-free DMEM medium. SEAP reporter levels in the cell supernatant were determined by QuantiBlue Assay 24 h later. Error bars represent the standard deviation of the mean of technical triplicates. The graph is representative of at least three independent experiments.

To characterize the effect of synthetic compounds FP13-17 on cells naturally expressing TLR4, we used the murine macrophage RAW 264.7-Blue cell line, which are murine RAW 264.7 macrophages with chromosomal integration of SEAP reporter construct described in the previous section. Also in this case, the compounds did not show agonist activity when provided alone to the cells, while they inhibited LPS-stimulated SEAP secretion in a dose-dependent manner (Fig. 8) and the potency in inhibiting TLR4 signal was similar for all synthetic compounds.

### *In vivo* experiments with compound FP13

To further investigate whether carboxylate-based lipid A mimetics are able to inhibit LPS-induced signaling *in vivo*, we injected C57BL/6 mice i.p. with 200 µg of compound FP13 followed by the LPS injection 30 min later. After 4 h mice were sacrificed and serum was analyzed for the presence of TNFα, which was strongly increased upon the LPS treatment (Supp. Inf. Fig. S11). However, in contrast to our cellular studies, FP13 did not affect LPS-induced serum TNFα levels. The reason for this was unclear and we hypothesized poor distribution and absorption of the compound upon i.p. injection.

We therefore also explored the antagonistic capacity of FP13 in a different *in vivo* model, where LPS and FP13 were both administered locally in the lungs. Intratracheal (i.t.) instillation of LPS is known to cause an acute inflammatory response in mice, characterized by transient infiltration of neutrophils into the airways^57,58^. However, also in this model, treatment with FP13 failed to suppress the LPS-induced effect (Supp. Inf. Fig. S12). These results demonstrate that FP13 is biologically inactive in mice when administered i.p. or i.t.

**Figure 9:**
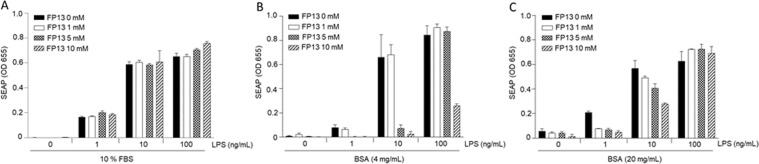
Presence of serum or BSA neutralizes the antagonistic activity of monosaccharide FP13 on LPS-induced TLR4 signaling in RAW 264.7-Blue cells. A-C. Cells were pre-incubated with the indicated concentrations of monosaccharide FP13 in DMEM supplemented with 10% FBS (**A**), 4 mg/mL BSA (**B**) or 20 mg/ml BSA (**C**). 30 min later cells were stimulated with 1, 10 or 100 ng/mL *E*. *coli* O111:B4 LPS. SEAP reporter levels in the cell supernatant were determined by QuantiBlue Assay 24 h later. Error bars represent the standard deviation of the mean of biological triplicates. The graph is representative of at least three independent experiments.

We next sought to determine the possible reason for the discrepancy in the observed effect of FP13 *in vitro* and *in vivo*. The plasma protein binding of therapeutic drugs is known to have a significant impact on their pharmacokinetics and pharmacodynamics^59^. It should be noted that the above described *in vitro* experiments were done with HEK293-Blue and RAW 264.7-Blue cells cultured in the absence of serum. Therefore, we first analyzed whether the presence of serum has an effect on the *in vitro* biological activity of FP13. Indeed, the antagonistic activity of FP13 was completely neutralized when tested on RAW 264.7-Blue cells incubated in DMEM supplemented with 10% FBS (Fig. 9A). Serum albumin is the major soluble protein constituent of blood with mouse albumin serum concentrations being around 20 mg/mL^60^. In order to evaluate whether the presence of serum albumin protein is sufficient to neutralize the *in vitro* activity of FP13, we repeated the RAW 264.7-Blue cell assay in serum-free conditions but with the addition of 4 or 20 mg/mL BSA. Similar to addition of complete serum, also addition of BSA strongly decreased the antagonistic activity of FP13 in a dose dependent manner (Fig. 9B,C). Together, these data indicate that binding of FP13 to proteins interferes with its biological activity and that binding of FP13 to albumin or other proteins present in mouse blood and the bronchoalveolar lung environment is most likely responsible for the absence of an antagonistic effect of FP13 *in vivo*.

## Discussion

This study presents a new series of monosaccharide lipid A mimetics containing two carboxylates replacing lipid A phosphates. The structure of these compounds is based on a glucosamine scaffold that has been functionalized with two succinate esters in C1 and C4, and therefore expose two carboxylic acids at a mutual distance similar to that of lipid A phosphates. Saturated and/or unsaturated fatty acid chains are attached to the C2 and C3 positions of the sugar.

The structure-based design of these compounds was done by molecular docking within the TLR4/MD-2 receptor and MD simulations. All compounds showed favorable predicted binding scores and interesting binding poses into hMD-2 that suggested their putative MD-2 binding properties and their possible activity as TLR4 modulators. Compounds were synthesized starting from commercial glucosamine through a divergent strategy from a common intermediate.

For the first time in the case of carboxylic-acid-based lipid A analogues, the interaction with functional hMD-2 receptor was directly measured by using a recombinant hMD-2 expressed in yeast (*P*. *pastoris*).

In a first set of *in vitro* experiments, we studied the SAR of these carboxylic-acid-based lipid A analogues (FP13-17). Four different binding experiments between synthetic compounds FP13-17 and hMD-2 were carried out. These were competition (displacement) experiments in which the synthetic glycolipids FP13-17 competed with biotin-LPS, with the fluorescent MD-2 ligand bis-ANS, and with anti-MD-2 antibody for MD-2 binding. SPR measurements allowed to analyze directly the binding between synthetic glycolipids and hMD-2. All binding experiments consistently provided a similar order of affinity among hMD-2 and synthetic molecules FP13-17.

The biological activity was assessed on cellular models expressing human and murine TLR4/MD-2. All molecules turned out to be inactive as agonists on human and murine TLR4. However, when administered with LPS, all the carboxylate-based lipid A mimetics were able to inhibit the LPS/TLR4 signal (antagonism) in both human (HEK293) and murine (RAW 264.7) TLR4-expressing cells with a similar potency, also similar to the previously characterized monosaccharide FP7.

FP13-17 present very similar IC_50_ in a range from 2.7 to 6.6 µM, which is a slightly higher value compared to the IC_50_ of FP7 (2.0 µM).

Although our results reveal FP13 and related compounds as strong antagonists of TLR4, their biological activity is abrogated by serum albumin, which most likely explains the absence of an antagonistic effect in the two different *in vivo* models of LPS activity. We propose that direct binding of FP13 to albumin or other proteins in serum and lungs neutralizes its biological activity *in vivo*. Definitive prove will require *in vitro* binding studies of FP13 with albumin. Together, these data demonstrate the need for additional chemical optimization or formulation of the current compounds.

The analysis of the cryo-TEM data of one compound of the series, FP15, indicates that this molecule mainly generates circular and homogeneous small unilamellar vesicles (SUV), with rather different size distributions, from 10 to 110 nm of diameter, with the presence of fusion events as well as open bilayers. The fact that FP15 forms stable aggregates is an important information for the pharmacological formulation of this type of compounds in the perspective of clinical development. Interestingly, the formation of large vesicles does not abrogate the biological activity, at least at cellular level.

MD simulation shows the self-assembly with formation of clusters. Succinate groups seems to stabilize complexes to form 3 main assemblies, “face-to-face” and “packed in parallel” clusters might be the reason for the strong stability of the membrane vesicles while “in-circle assembly” cluster suggests a high permeability to water.

The main achievement of this is work is demonstrating that this type of new carboxylate-based monosaccharidic lipid A analogues strongly interact with co-receptor MD-2 displacing the natural ligand LPS and forming stable complexes.

MD-2 strong interaction and binding is very likely the main mechanism of TLR4 antagonism for this series of compounds.

Concerning the molecular design, we confirm that the carboxylic acid represents an effective bioisostere of phosphoric acid and can be used in this type of lipid A mimetics retaining MD-2 molecular recognition and binding. The replacement of phosphates with carboxylates is an important step towards druggability, as carboxylates are metabolically more stable and easier to synthesize. On the contrary, the presence of an unsaturation in the fatty acid chains seems to play a minor role since the *in vitro* affinity for the purified hMD-2 receptor and the activity on cells is almost identical for saturated (**FP17**) and unsaturated (FP13-16) compounds.

In conclusion, the novelty and importance of our work is the description of new carboxylate-based monosaccharidic lipid A analogues that strongly interact with the co-receptor MD-2, displacing the natural ligand LPS and forming stable complexes. The interaction and binding with MD-2 is most likely the main mechanism of TLR4 antagonism for this series of compounds. FP13-17 showed to bind MD-2, are active on cells as TLR4 antagonists in the absence of serum proteins and are non-toxic at a concentration of 10 μM. The lack of *in vivo* activity is probably due to strong interaction with serum albumin and further structure optimization is in progress to improve the pharmacokinetic of these promising hit compounds.

The biological activity of this panel of compounds as TLR4 antagonists, combined with the tendency to spontaneously form vesicular aggregates, make this type of carboxylate-based lipid A analogues interesting in the perspective to exploit the self-assembled aggregates structures to modulate their pharmacokinetic and biodistribution properties in biomedical and pharmacological contexts^61–64^.

## Methods

All methods were performed in accordance with the relevant guidelines and regulations. Methods for the chemical synthesis of monosaccharides and computational methods are provided at the Supp. Inf.

### Preparation of recombinant MD-2 in *Pichia pastoris* and purification

MD-2 was produced in *Pichia pastoris*, analyzed by SDS–PAGE and its biological activity tested on 293/hTLR4a cells as described before^51^.

### Antibody-sandwich ELISA for the detection of binding of compounds to MD-2

The method of antibody-sandwich ELISA for the detection of the binding of compounds to MD-2 was modified from a previous study^51^. A microtiter plate was coated overnight at 4 °C with 100 μL/well of 5 μg/mL of chicken polyclonal anti-hMD-2 antibodies, diluted in 50 mM Na_2_CO_3_ buffer, pH 9.6 and blocked with 1% BSA in PBS. After washing, 1 μM hMD-2 with tested compounds was added and incubated for 2 hours. 0.1 μg/mL mouse anti-hMD-2 MAb (9B4) and 0.1 μg/mL goat anti-mouse IgG conjugated with HRP in PBS were added, followed by detection at 420 nm after the addition of 100 μL of ABTS (Sigma). Chicken anti-hMD-2 polyclonal antibodies were prepared against recombinant hMD-2 by GenTel (Madison, WI, USA), monoclonal mouse anti-hMD-2 9B4 antibodies were from eBioscience (San Diego, CA, USA), and secondary goat anti-mouse IgG conjugated with horseradish peroxidase were from Santa Cruz Biotechnology (Santa Cruz, CA, USA).

### Fluorescence spectroscopy assay

Fluorescence was measured on Perkin Elmer fluorimeter LS 55 (Perkin Elmer, UK) as previously described^51^. All measurements were done at 20 °C in a 5 × 5 mm quartz glass cuvette (Hellma Suprasil, Müllheim, Germany). hMD-2 protein (200 nM) and 1,1′-Bis(anilino)−4,4′-bis (naphthalene)−8,8′ disulfonate (bis-ANS, 200 nM) were mixed and incubated until reaching stable relative fluorescence units (RFUs) emitted at 420–550 nm under excitation at 385 nm. Compounds, at different concentrations, were then added, followed by relative fluorescence unit (RFU) measurement at 420–550 nm.

### LPS displacement assay

The ability of the compounds to displace LPS from hMD-2 hydrophobic pocket was determined by ELISA. A microtiter plate was coated overnight at 4 °C with 100 μL/well of 5 μg/mL of chicken polyclonal anti-hMD-2 antibodies, diluted in 50 mM Na_2_CO_3_ buffer, pH 9.6 and blocked with 1% BSA in PBS. After washing, 1 μM of hMD-2 with biotin-labeled LPS was added and incubated for 2 hours. After washing, the compounds were added at different concentration and incubated for 1.5 hours. After washing, 0.5 μg/mL HRP-conjugated streptavidin (Sigma) in PBS was added, followed by detection at 420 nm after the addition of 100 μL ABTS (Sigma). Chicken anti-hMD-2 polyclonal antibodies were prepared against recombinant hMD-2 by GenTel (Madison, WI, USA).

### Surface plasmon resonance (SPR) analysis

The binding affinity of the compounds to recombinant hMD-2 was determined using a Biacore X100 with an NTA sensor chip (Biacore, GE Healthcare, Uppsala, Sweden). Briefly, 0.5 μM hMD-2 (in 50 mM TRIS, 150 mM NaCl, 0.5% Tween 20, pH 7.5) was immobilized onto the sensor chip previously activated with 1-min. pulse of 10 mM NiSO_4_. First flow cell was used as a reference surface to control non-specific binding. Both flow cells were injected with the analyte (in PBS, 5% DMSO, 5% EtOH, pH 7.5) at a flow rate of 10 μL/min at 25 °C in increasing concentrations. The data were analyzed with Biacore Evaluation software. K_D_ values were calculated by global fitting of the equilibrium binding responses from various concentrations of analytes using a 1:1 Langmuir binding model.

### HEK-Blue hTLR4 cells assay

HEK-Blue hTLR4 cells (InvivoGen) were cultured according to manufacturer’s instructions. Briefly, cells were cultured in DMEM high glucose medium supplemented with 10% fetal bovine serum (FBS), 2 mM glutamine, antibiotics and 1 × HEK-Blue Selection (InvivoGen). Cells were detached using a cell scraper, counted and seeded in a 96-well multiwell plate at a density of 4 × 10^4^ cells per well. After overnight incubation (37 °C, 5% CO2, 95% humidity), supernatants were replaced with new medium w/o FBS supplemented by the compound to be tested and incubated for 16 hours. For the antagonism evaluation, cells were stimulated with LPS (100 ng/mL, *E*. *coli* O55:B5, Sigma-Aldrich) after compounds pre-treatment and incubated for 16 hours as above. The SEAP-containing supernatants were collected and incubated with *para*-nitrophenyl phosphate (pNPP) for 2−4 h in the dark at room temperature. The wells optical density was determined using a microplate reader (labtech LT-4000) set to 405 nm.

### MTT cell viability assay

HEK-Blue hTLR4 cells were grown in DMEM supplemented with 10% FBS, 2 mM glutamine and antibiotics. Cells were seeded in 100 μL of DMEM w/o Phenol Red at a density of 4 × 10^4^ cells per well and incubated overnight (37 °C, 5% CO_2_, 95% humidity). Cells were treated with the higher dose of compound used in the previous experiments (10 μM) and incubated overnight. MTT solution (5 mg/mL in PBS) was added to each well and after 3 hours incubation, HCl 0.1 N in 2-propanol solution was used to dissolve formazan crystals. Formazan concentration was determined by measuring the absorbance at 570 nm.

### Experiments on RAW 264.7-Blue cells

Murine RAW 264.7-Blue cells (Invivogen) derived from the murine RAW 264.7 macrophages and stably expressing an NF-κB-inducible secreted alkaline phosphatase (SEAP) reporter gene were cultured in Dulbecco’s modified Eagle’s medium (DMEM), supplemented with 10% Fetal bovine serum (FBS), L-Glutamine (2 mM) and sodium pyruvate (0.4 mM), in the presence of selection antibiotic Zeocin (200 μg/mL). *E*. *coli* O111:B4 LPS was purchased from Invivogen. Bovine serum albumin (BSA) was purchased from Sigma-Aldrich. Compounds FP13-17 were reconstituted in DMSO/ethanol to provide a 10 mM stock solution. Further dilutions were made in cell culture medium so that the final amount of DMSO in the cell culture did not exceed 0.05%. C57BL6/J mice were obtained from Janvier. All animal experiments were approved by the animal ethical committee of Ghent University, Faculty of Sciences. All methods were performed in accordance with the relevant guidelines and regulations. *Salmonella enterica* LPS used for *in vivo* studies was purchased from Sigma-Aldrich.

#### Biological activity assay

5 × 104 RAW 264.7-Blue cells were pre-treated with compounds FP13-17 in a total volume of 200 µl serum-free DMEM supplemented with L-Glutamine (2 mM) and sodium pyruvate (0.4 mM). In some experiments DMEM was supplemented with 10% FBS or BSA at the concentrations of 4 mg/mL and 20 mg/mL. 30 min. later, cells were stimulated with *E*. *coli* O111:B4 LPS, as indicated. 24 h later cell supernatants were analyzed for SEAP production by the QUANTI-Blue Assay (Invivogen).

### Cryo-TEM sample preparation

All samples were prepared in 10 mM buffer phosphate (pH 7.4) with 16% of DMSO. *Cryo-TEM* data were collected on a JEM-2200FS/CR transmission electron microscope (JEOL, Japan), equipped with an UltraScan 4000 SP (4008 × 4008 pixels) cooled slow-scan CCD camera (GATAN, UK). Three microliters of the compound were vitrified on Quantifoil 2/2 grids, using Vitrobot (FEI) and were analyzed at nitrogen liquid temperature with a TEM operated at 200 kV in low dose conditions.

### Ethical Approval

All animal experiments were approved by the Ethical Committee for Animal Experimentation of Ghent University – Faculty of Sciences.

## Supplementary information

Supp Info
