# Supplementary material for: Novel carboxylate-based glycolipids: TLR4 antagonism, MD-2 binding and self-assembly properties
Source: Sci Rep. 2019 Jan 29;9:919. doi: 10.1038/s41598-018-37421-w (PMC6351529; doi:10.1038/s41598-018-37421-w)
Supplement: Supplementary file 1 — Supp Info [file 41598_2018_37421_MOESM1_ESM.doc]

# Supporting information

Novel carboxylate-based glycolipids: TLR4 antagonism, MD-2 binding and self-assembly properties

Florent Cochet^1^, Fabio A. Facchini^1^, Lenny Zaffaroni^1^, Jean-Marc Billod^2^, Helena Coelho^3^, Aurora Holgado^4^, Harald Braun^4^, Rudi Beyaert^4^, Roman Jerala^5^, Jesus Jimenez-Barbero^6^, Sonsoles Martin-Santamaria^2^, Francesco Peri^1*^

*^1^* Department of Biotechnology and Biosciences, University of Milano-Bicocca, Piazza della Scienza, 2; 20126 Milano (Italy).

*^2^* Department of Structural and Chemical Biology, Centro de Investigaciones Biologicas, CIB-CSIC, C/Ramiro de Maeztu, 9, 28040 Madrid, Spain.

^3^ Molecular Recognition & Host−Pathogen Interactions Programme, CIC bioGUNE, Bizkaia Technology Park, Building 801A, 48170 Derio (Spain); UCIBIO, REQUIMTE, Departamento de Química, Faculdade de Ciências e Tecnologia, Universidade Nova de Lisboa, 2829-516 Caparica (Portugal); Department of Organic Chemistry II, Faculty of Science & Technology, University of the Basque Country, 48940 Leioa, Bizkaia (Spain).

*^4^* Unit for Molecular Signal Transduction in Inflammation VIB-UGent Center for Inflammation Research, VIB Technologiepark 927, 9052 Zwijnaarde, Ghent (Belgium); Department of Biomedical Molecular Biology, Ghent University Technologiepark 927, 9052 Zwijnaarde, Ghent (Belgium).

*^5^* Department of Biotechnology, National Institute of Chemistry, Hajdrihova 19, 1000 Ljubljana (Slovenia).

*^6^* Molecular Recognition & Host−Pathogen Interactions Programme, CIC bioGUNE, Bizkaia Technology Park, Building 801A, 48170 Derio (Spain); Department of Organic Chemistry II, Faculty of Science & Technology, University of the Basque Country, 48940 Leioa, Bizkaia (Spain); Ikerbasque, Basque Foundation for Science, Maria Diaz de Haro 13, 48009 Bilbao (Spain).

Molecular modeling

*Structure construction and refinement.* The 3D structures of ligands **FP13**, **FP14**, **FP15**, **FP16** and **FP17** were built with PyMOL using the 3D structure of **FP7**, previously reported,^50^ as template. All structures were then optimized at the Hartree-Fock level. For the human TLR4/MD-2 system, we used a computational 3D model in antagonist conformation previously modeled by us. Information about the construction of the 3D coordinates of this TLR4/MD-2 model can be found here.^47^

The 3D structures of ligands **FP13**, **FP14**, **FP15**, **FP16** and **FP17** were built with PyMOL using the 3D structure of **FP7**, previously reported,^50^ as template. All structures were then optimized at the Hartree-Fock level. For the human TLR4/MD-2 system, we used a computational 3D model in antagonist conformation previously modeled by us. Information about the construction of the 3D coordinates of this TLR4/MD-2 model can be found here.^47^

*Parameters Derivation.* The missing parameters for the MD simulations were calculated using the standard Antechamber procedure for Amber14.^63^ Briefly, ligand structures, already refined at the AM1 level of theory, were optimized and their atomic partial charges were calculated with Gaussian09/e1^64^ at the Hartree−Fock level (HF/6-31G* Pop = MK iop(6/33 = 2), iop(6/42 = 6)). Partial charges were derived and formatted for AmberTools15 and Amber14 with Antechamber, assigning the general AMBER force field (GAFF) atom types. The atom types of the atom constituting the saccharide ring were changed to the GLYCAM force field atom types.^65^ The GAFF parameters for the phosphate group were modified as reported here.^50^

*Docking calculations of ligands FP13-17.* AutoDock Vina 1.1.2 ^48^ was used for the docking of the ligands in the human TLR4/MD-2 antagonist model and AutoDock 4.2 ^49^ was used to re-dock some selected poses. In AutoDock 4.2, the Lamarckian evolutionary algorithm was chosen, and all parameters were kept default except for the number of genetic algorithm runs that was set to 200 to enhance the sampling. AutoDockTools 1.5.6^49^ was used to assign the Gasteiger-Marsili empirical atomic partial charges to the atoms of both the ligands and the receptor. Nonpolar hydrogens were merged for all the ligands. The structure of the receptor was always kept rigid, whereas the structure of the ligand was set flexible. Regarding the docking boxes, spacing was set to the default value of 1 Å for Vina, and of 0.375 Å for AutoDock. The size of the box was set to 33.00 Å in the x-axis, 40.50 Å in the y-axis and 35.25 Å in the z-axis, and the center of the box was located equidistant to the center of mass of residues Arg90 (MD-2), Lys122 (MD-2) and Arg264 (TLR4).

*Molecular dynamics (MD) simulations.* MD simulations of selected docked TLR4/MD-2/ligand complexes were performed with amber14 and amber16. The proteins were described by the ff14SB all-atom force field,^66^ the saccharide core of the ligands by the GLYCAM_06j-1 force field^65^ and the other moieties of the ligands were parametrized with the GAFF force field. The simulation box was designed such as the edges are distant of at least 10 Å of any atoms. The system was solvated with the TIP3P water molecules model. Na^+^ and Cl^-^ ion were added to counterbalance the eventual charges of the protein-ligand systems. All the simulations were performed with the same equilibration and production protocol. First, the system was submitted to 1000 steps of steepest descent algorithm followed by 7000 steps of conjugate gradient algorithm. A 100 kcal mol^-1^ A^-2^ harmonic potential constraint was applied on the proteins and the ligands. In the subsequent steps, the harmonic potential was progressively lowered (respectively to 10, 5, 2.5 and 0 kcal mol^-1^ A^-2^) for 600 steps of conjugate gradient algorithm each time. Next, the system was heated from 0 K to 100 K by a Langevin thermostat in the canonical ensemble (NVT) under a 20 kcal.mol^-1^.A^-2^ harmonic potential restraint on the proteins and the ligand. Finally, the system was heated up from 100 K to 300 K in the Isothermal-isobaric ensemble (NPT) under the same restraint condition than the previous step, followed by a simulation of 100 ps in which all harmonic restraints were removed. At this point the system was ready for the production run, which was performed using the Langevin thermostat in the NPT ensemble, at a 2 fs time step.

*Molecular dynamics simulations of FP15 spontaneous assembly.* In the experiment **FP15** was added to a phosphate buffer (PB) and 5% DMSO preparation, we thus assume the counter ions to be Na^+^. To create the starting structure for the self-assembly 128 molecules of **FP15** were randomly distributed in a cubic box of 80 Å^3^ with Packmol.^67^ The same starting random spatial distribution was simulated at different temperatures (ranging from 300K to 400K) in order to minimize the simulation time while increasing the assembly speed. Within the NpT ensemble pressure was handled both isotropically (to trigger the self-assembly) and anisotropically (for the production run of the bilayer). A total of 5 sets of simulations were conducted. A first simulation in isotropic conditions at 300K for 180 ns then anisotropic conditions at 303K for 120 ns (total of 300 ns), a second one in isotropic conditions at 310K for 200 ns, a third one in isotropic conditions at 350K for 150 ns and then 850 ns of simulation in anisotropic conditions and finally two independent simulations at 400K, one in anisotropic condition for 500ns and another one in isotropic conditions for 400ns. More explanations about the different simulations are given in SI. The MD protocol used is the same than the one described for the simulations of the docked poses at the exception that temperature was changed accordingly to the desired one and pressure during the production phase was switched from isotropic to anisotropic as mentioned above.


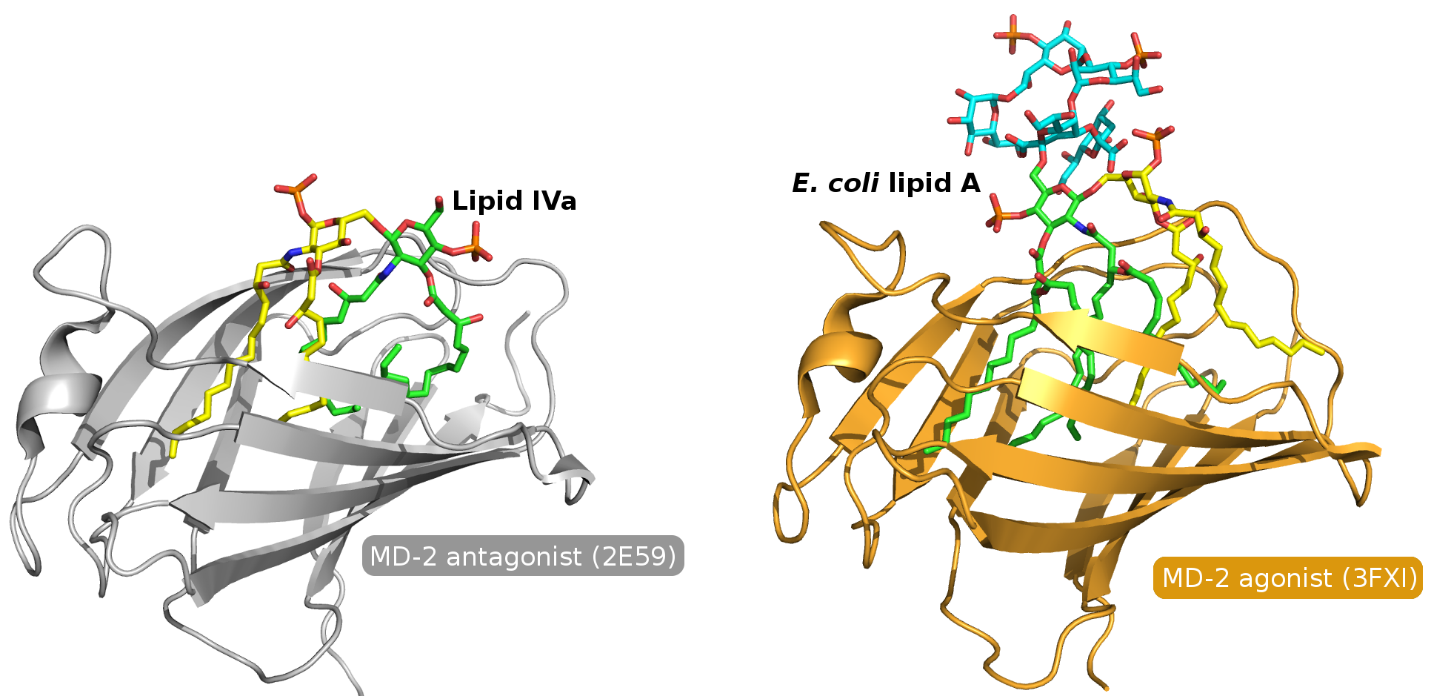
Figure S1**.** On the left: representation of type A (antagonist-like) binding mode as known from lipid IVa in PDB ID 2E59. On the right: representation of type B (agonist-like) binding mode as for *E. coli* lipid A in PDB ID 3FXI.

Small Unilamellar Vesicles formation: computational study**.**

MD simulations conducted at 300K, 303K and 310K failed to reach phase separation, in which the lipids would continuously span two dimensions interacting one with another and be separated by solvent in the third. This might be due to a too short simulation time, such system might require longer timeframes to evolve to a more stable equilibrium state. It also appears that the lipids have a tendency to freeze, which could be due to the set of parameters selected, the temperature, being controlled by a Langevin thermostat, should be distributed evenly throughout the three dimensional space. At 350K and 400K the system reached phase separation. However at 400K, in our attempt at starting the MD simulation under anisotropic conditions, the bilayer did not form in the two dimensions parallel to any face of the box (rather in a diagonal fashion), causing two dimensions of the box to progressively collapse over time. The other simulation (a 400-ns simulation under isotropic conditions) also reached good phase separation (data not shown). Among the two simulations to reach phase separation we selected the 350K one over the 400K one for performing an in-depth analysis, considering that the one with the lowest temperature is more likely to better represent the reality. In turn, the selected simulation was run for 1 µs, in which 150 ns were run under isotropic conditions and 850 ns under anisotropic conditions, all at 350K. In most of the MD simulations we noted that under anisotropic conditions the system once already pre-equilibrated has a tendency to evolve very slowly.

| **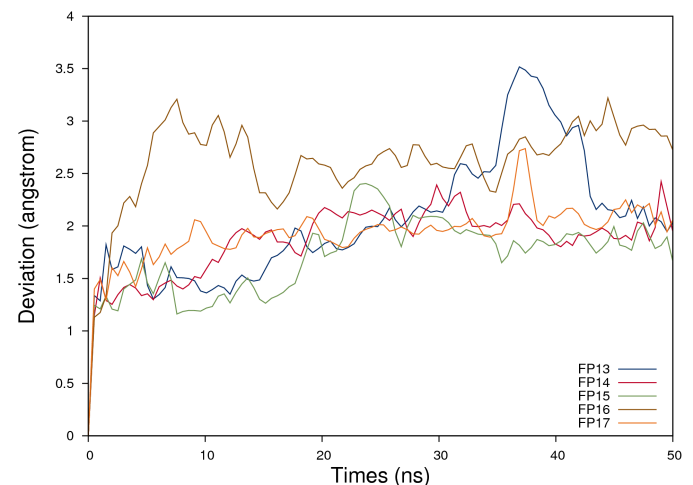** | **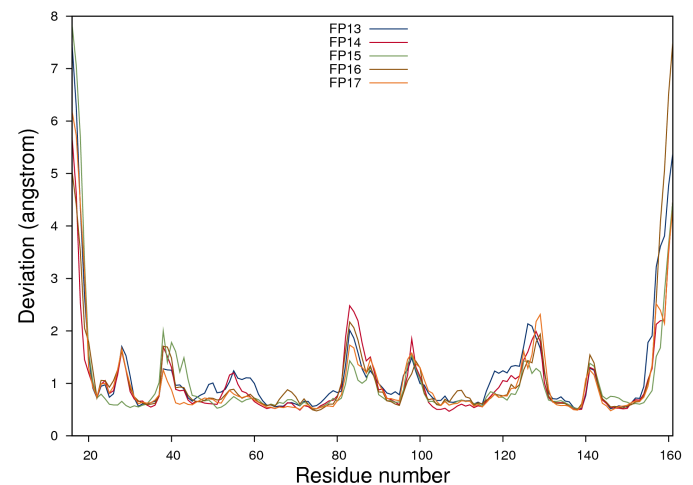** |
| --- | --- |

Figure S2**.** Molecular dynamics simulations of the TLR4/MD-2 system in complex with ligands FP13, FP14, FP15, FP16 and FP17. A) RMSD of the MD-2 backbone over time. B) RMS fluctuations per residues of MD-2.

| 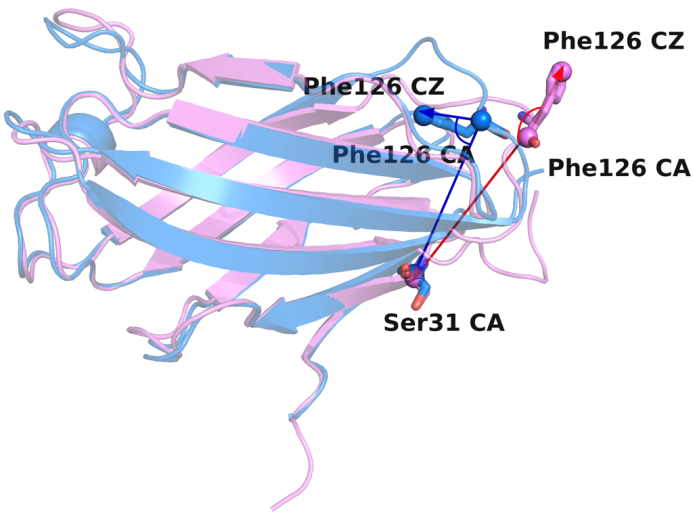 | 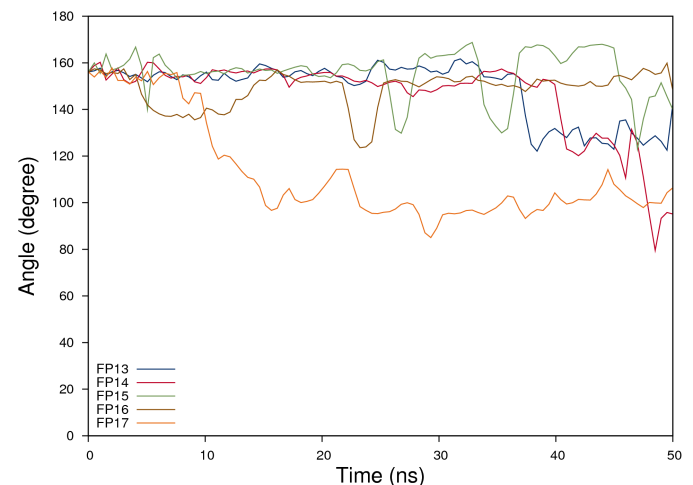 |
| --- | --- |

Figure S3**.** On the left: representation of two vectors, within MD-2, starting both from the alpha-carbon of residue Phe126 to, respectively, the zeta-carbon of the same residue and the alpha-carbon of residue Ser21. Agonist MD-2 from PDB-ID 2E59 and antagonist MD-2 from PDB-ID 3FXI are represented in semi-transparent blue and pink cartoons respectively. On the right: the angle between the two vectors defined in A is plotted within MD2 of each MD simulations.

| 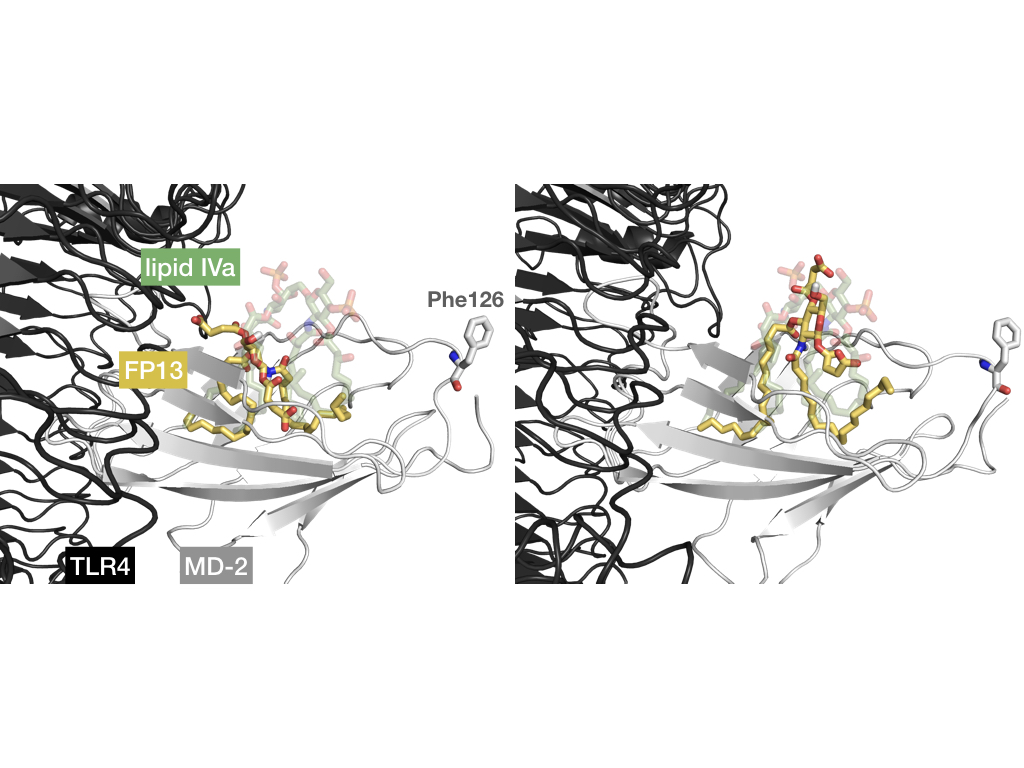 |  |
| --- | --- |

Figure S4**.** TLR4 and MD-2 are respectively represented in black and grey cartoon. **FP13** and lipid IVa (partially transparent) are depicted in CPK colored sticks with carbon atoms respectively colored in yellow and green. MD-2 Phe126 is in sticks. On the left: docked pose (t=0ns). On the right: end of the simulation (t=50ns).


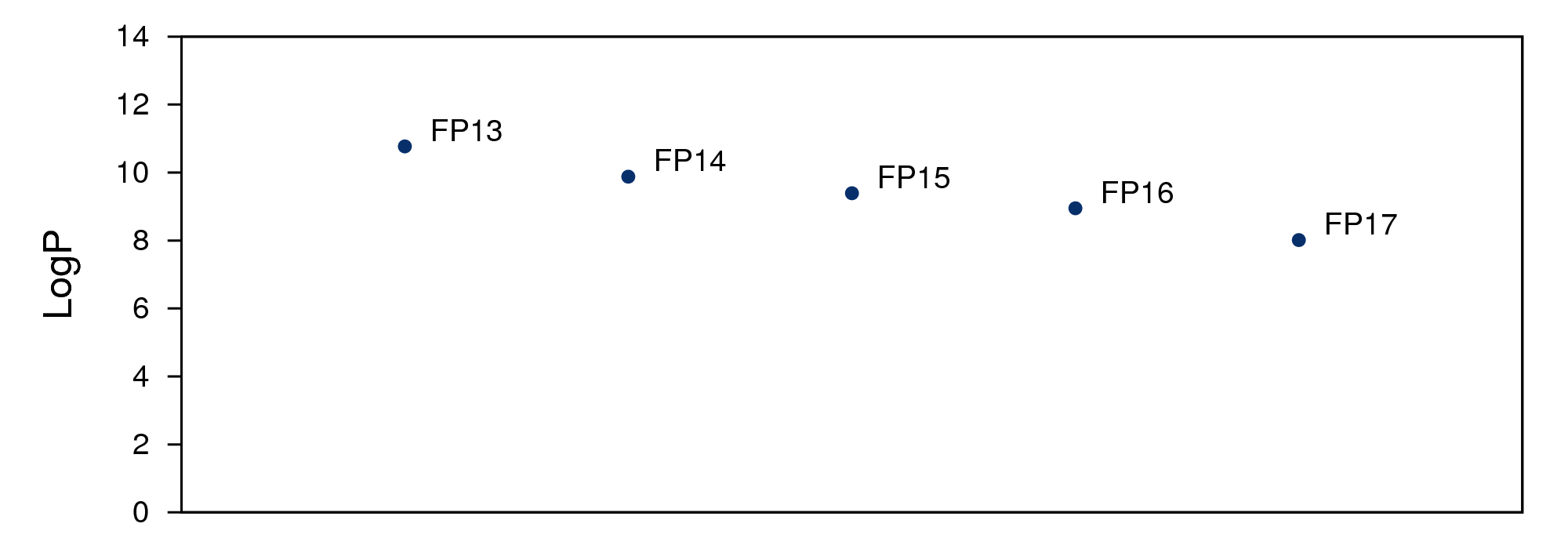


Figure S5**.** Computed logP values for compounds FP13, FP14, FP15, FP16 and FP17, as calculated in Maestro.[[1]](#footnote-2)

**Chemistry. Synthesis of monosaccharides**

Synthesis of **FP13-17** series was achieved following the divergent synthetic strategy depicted in the Scheme 1. D-glucosamine was treated with sodium azide and triflic anhydride to obtain the azido derivative at position C2. Positions C4 and C6 were protected as 4,6-di-O-benzylidene and position C1 was protected as *tert*-butyldimethylsilyl ether (TBS) to obtain compound **1** as a common intermediate for all compounds (Figure S6).


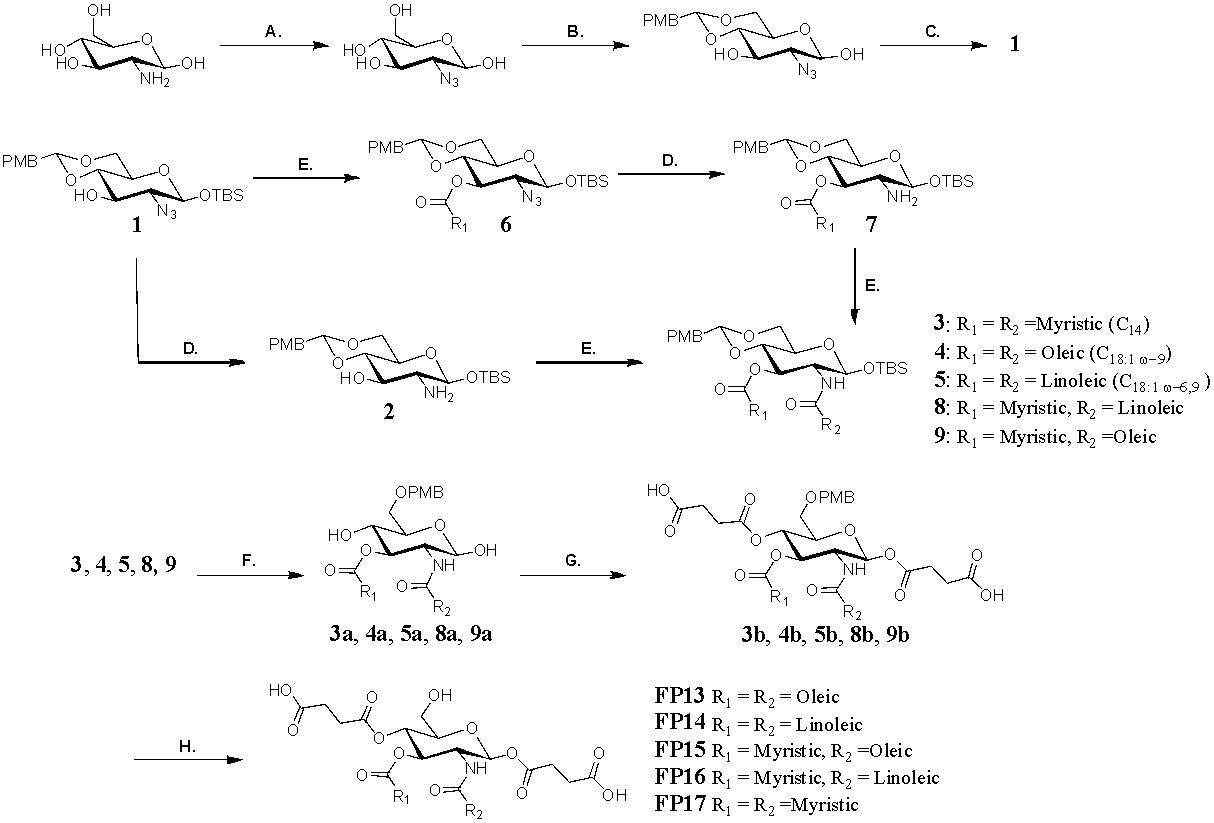


**Figure S6**. Synthesis of compounds **FP13-17**. Reagents and conditions:  **A.** i) NaN_3_, Tf_2_O, Pyr. ii) CuSO_4_, Et_3_N, H_2_O, 0°C to r.t.; **B.** ADMA, p-TsOH.H_2_O (cat.), DMF, (80%); **C.** TBDMSCl, Imidazole, DCM, (75%);  **D.** PPh_3_, H_2_O, THF, 60°C, (85%); **E.** Myristic, Oleic or Linoleic acid, EDC, DMAP, DCM, (60%); **F.** NaBH_3_CN, HCl, 4 Å MS, THF, (62%); **G.** Succinic anhydride, DMAP, Et_3_N, DCM, (80%); **H.** TFA, DCM, 0°C to r.t., (75%).

For the synthesis of **FP13**, **FP14** and **FP17** having in glucosamine positions C2 and C3 two identical chains, **1** was treated with triphenylphosphine (PPh_3_) in THF/H_2_O to hydrolyze the azide into an amine thus obtaining **2**. C2 and C3 positions were then esterified with myristic, oleic or linoleic acid in the presence of the 1-ethyl-3-(3-dimethylaminopropyl)carbodiimide (EDC) as a condensing agent and a catalytic amount of dimethyl aminopyridine (DMAP) in dichloromethane giving, respectively, compounds **3**, **4** and **5**.

For the synthesis of compounds with two different chains in C2 and C3, azido-glucose **1** was esterified at position C3 using myristic acid, in the presence of DMAP and EDC in dichloromethane, to give intermediate **6**. After transformation of the azide into amine (**7**) and subsequent esterification with linoleic or oleic acid was obtained, respectively, compounds **8** (R^1^=myristic; R^2^=linoleic) and **9** (R^1^=myristic; R^2^=oleic).

Intermediates **3**, **4**, **5**, **8** and **9** were transformed into final products through the same reactions sequence. The regioselective opening of benzylidene with sodium cyanoborohydride and hydrochloric acid followed by reaction with succinic anhydride and deprotection of the para-methoxybenzyl group in position C6 by acid treatment (trifluoroacetic acid, TFA), afforded final products **FP13-17**.

**Experimental procedures.**

*General.* Commercially available reagents and solvents were used without further purification. Reactions were monitored by thin-layer chromatography (glass plates coated with silica gel 60 F254 from Merck KGaA). Products were purified with column chromatography on flash Silica gel 60A (40-63u from Fluorochem). ^1^H and ^13^C NMR spectra were recorded at room temperature in deuterated solvents on a Varian Mercury 400MHz Spectrometer. Chemical shifts (δ) are reported in parts per million (ppm) relative to TMS as internal standard or relative to the solvent [^1^H: δ(CDCl_3_) = 7.26 ppm, δ(CD_3_OD) = 3.31 ppm, δ(acetone-d_6_) = 2.05 ppm; ^13^C: δ(CDCl_3_) = 77.16 ppm, δ(CD_3_OD) = 49.00 ppm, δ(acetone-d_6_) = 29.84 ppm]. Electrospray ionization ESI mass spectra were acquired on an AB SCIEX 2000 QTRAP LC/MS/MS with ESI source, mechanical pump and Analyst Data System.

1. *Azide protection:* In a two-necked flask filled with Argon was added NaN_3_ (1.2 eq.) in Pyridine (2 ml/mmol). Reaction was cooled down in an ice bath and Tf_2_O (1.4 eq.) was added dropwise. The reaction was stirred in ice during 3h. In a second flask was added glucosamine (1 eq.) in pyridine/H_2_O (1:3, 1 ml/mmol). The reaction was cooled down to 0°C, were added CuSO_4_ (0.065 eq.), TEA (2 eq.) and the reaction was stirred during 30 min. The first preparation was transferred in the second-one and the mixture was stirred during 24h at r.t. Reaction was monitored by TLC (AcOEt/MeOH/H_2_O 7:3:0.1, R_f_ = 0.6). Then, half of the solvent was evaporated under vacuum, toluene was added (1:1) and co-evaporated under vacuum several times until a white precipitate appears. Product was used without further purification in the successive step, assuming 100 % yield.
2. *Benzylidene formation*: Azide protected glucosamine (1 eq.) from the previous reaction was dissolved in DMF_Dry_ (~1 ml/mmol). Anisaldehyde dimethyl acetal (ADMA, 1.1 eq.) and para-toluene-sulfonic acid (*p*-TsOH, 0.02 eq.) were added and the reaction was performed on the rotavapor (60°C, 250 mbar) during 30 min. Reaction was monitored by TLC (AcOEt /EtP 6:4, R_f_ = 0.7). Then, reaction was quenched with TEA (0.04 eq.) and evaporated under vacuum. Product was purified on flash column chromatography (AcOEt/EtP 1:1) to afford the product as a yellow powder. Global yield of the two reactions: 80%.
3. *Anomeric protection by TBDMS:* Compound obtained after procedure *B*. (1 eq.) was dissolved in DCM_Dry_ (2 ml/mmol) under argon and cooled down until -10°C. Tert-butyldimethylsilyl chloride (1.2 eq.) was added together with imidazole (2.5 eq.). The reaction was followed by TLC (EtP/AcOEt 8:2, R_f_ = 0.9). Then, H_2_O (0.2 ml/mmol) was added and the mixture was diluted with DCM (2 ml/mmol) and washed with H_2_O (2 ml/mmol). The aqueous layer was extracted with DCM (2x 1 ml/mmol), and the combined organic layers were dried with Na_2_SO_4_ and concentrated under vacuum. The residue was purified on flash column chromatography (AcOEt/EtP 1:9) to afford the product as a slightly yellow oil with 75% yield.
4. *Azide deprotection:* The suitable azide protected carbohydrate from procedure *C* (1 eq.) was dissolved in THF (10 ml/mmol) and PPh_3_ (3 eq.) was added. The reaction was stirred 30 min at r.t and, then, heated to 60°C. At this point, H_2_O (1 ml/mmol) was added. The reaction was followed by TLC (AcOEt/EtP 2:8, R_f_ = 0.2). Then, the mixture was dried under vacuum and purified on flash column chromatography (AcOEt/EtP 1:9) to afford the desired product as a slightly yellow oil with 85% yield.
5. *Acylation (for double acylation):* Into a dry flask filled with argon were dissolved the suitable fatty acid chain (4 eq.) and EDC (6 eq.) in DCM (5 ml/mmol). The reaction was stirred 10 min at r.t. In a second dry flask filled with argon was dissolved the carbohydrate from protocol *D* (1 eq.) in DCM (5 ml/mmol) and, then, transferred into the first flask. The mixture was stirred 5 min before adding DMAP (1 eq.) and monitored by TLC (AcOEt/EtP 2:8, R_f_ = 0.7). Then, the mixture was diluted with DCM (5 ml/mmol) and washed with saturated NaHCO_3_ and brine. The organic phase was dried over NaSO_4_, filtrated and evaporated under vacuum before being purified on flash column chromatography (AcOEt/EtP 0.5:9.5) to afford the desired product as a slightly yellow oil with 60% yield.
6. *One-pot regio-selective opening of PMP benzylidene and anomeric silyl deprotection:* Product of procedure *E*. (1 eq.) was dissolved in dry THF (45 ml/mmol). NaBH_3_CN (15 eq.) and 4 Å molecular sieves (200 mg/mmol) were added. Mixture was stirred at r.t. for 2 h and, then, cooled to 0°C. A solution of HCl (1 M in dioxane, 18 eq.) was added dropwise and the mixture was stirred for 1h at 0°C and another 3h at r.t. (AcOEt/EtP 1:1, R_f_ = 0.3). Et_3_N (0.5 mL/mmol) was added to terminate the reaction. Molecular sieve was filtered off through Celite and washed with ether. The filtrate and wash were combined and washed with saturated NaHCO_3_ and brine, dried over Na_2_SO_4_ and finally evaporated under vacuum. The residue was purified on flash column chromatography (AcOEt/EtP 4:6) to afford the desired product as a colorless oil with 62% yield.
7. *1,4 -oxobutanoic acid formation:* To a solution of the compound from procedure *F* (1 eq.) and DMAP (6 eq.) in DCM (50 ml/mmol) was added succinic anhydride (6 eq.). The mixture was stirred overnight and monitored by TLC (DCM/MeOH 5% + 1% formic acid, R_f_ = 0.4). Then, the mixture was concentrated under vacuum and purified on flash column chromatography (DCM/MeOH 2% + 1% formic acid) to afford the desired product as a slightly yellow oil with 80% yield.
8. *PMB Deprotection:* To a solution the compound from procedure *G* (1 eq.) in DCM (100 ml/mmol) at 0°C, was added TFA (10 eq.). The reaction was stirred at room temperature and monitored by TLC (DCM/MeOH 5% + 1% formic acid, R_f_ = 0.2). Then, treated by saturated NaHCO_3_ and extracted twice by DCM (20 ml/mmol). The organic phase was washed with brine, dried over Na_2_SO_4_, filtrated and solvent was evaporated under vacuum. The residue was forwarded to column chromatography (DCM/MeOH 2% to 10% + 1% formic acid) to afford the final product as a brown oil with 75 % yield.

**Compounds characterization:**

*(4aR,6S,7R,8R,8aS)-7-azido-6-((tert-butyldimethylsilyl)oxy)-2-(4-methoxyphenyl)*

*hexahydro-pyrano[3,2-d][1,3]dioxin-8-ol (****1****):* **^1^H NMR (400 MHz, CDCl_3_) δ** 7.39 (d, J = 8.7 Hz, 2H, 2*HAr), 6.89 (d, J = 8.5 Hz, 2H, 2*HAr), 5.48 (s, 1H, CHPMB), 4.64 (d, J = 7.6 Hz, 1H, H1), 4.27 (dd, J = 10.5, 4.9 Hz, 1H, H4), 3.84 – 3.67 (m, 4H, OMe, H6a), 3.58 (dt, J = 28.0, 9.2 Hz, 2H, H3, H6b), 3.46 – 3.37 (m, 1H, H5), 3.32 (t, J = 8.5 Hz, 1H, H2), 0.94 (s, 9H, tBu), 0.16 (s, 6H, 2*Me-Si). **^13^C NMR (400 MHz, CDCl_3_) δ** 159.20, 129.95, 128.81, 113.36, 102.04, 100.73, 81.24, 70.68, 67.93, 64.74, 56.04, 49.77, 25.76 – 25.55, 18.59, -2.89, -3.29.

*(4aR,6S,7R,8R,8aS)-7-amino-6-((tert-butyldimethylsilyl)oxy)-2-(4-methoxyphenyl)*

*hexahydro-pyrano[3,2-d][1,3]dioxin-8-ol (****2****):* **^1^H NMR (400 MHz, CDCl_3_) δ** 7.41 (d, J = 8.6 Hz, 2H, 2*HAr), 6.88 (d, J = 8.6 Hz, 2H, 2*HAr), 5.48 (s, 1H, CHPMB), 4.56 (d, J = 7.4 Hz, 1H, H1), 4.25 (dd, J = 10.4, 4.8 Hz, 1H, H4), 3.84 – 3.71 (m, 4H, OMe, H6a), 3.64 (t, J = 9.2 Hz, 1H, H3), 3.54 (t, J = 8.9 Hz, 1H, H6b), 3.44 (dt, J = 14.1, 7.1 Hz, 1H, H5), 2.75 (t, J = 8.6 Hz, 1H, H2), 0.92 (s, 9H, tBu), 0.13 (s, 6H, 2*Me-Si). **^13^C NMR (400 MHz, CDCl_3_) δ** 159.21, 129.98, 128.76, 113.62, 102.06, 100.22, 80.59, 71.18, 67.93, 64.74, 60.74, 56.04, 25.62 – 25.58, 18.59, - 4.79, -5.26.

*(4aR,6S,7R,8R,8aS)-6-((tert-butyldimethylsilyl)oxy)-2-(4-methoxyphenyl)-7-tetradecan*

*amidohexahydropyrano[3,2-d][1,3]dioxin-8-yl tetradecanoate (****3****):* **^1^H NMR (400 MHz, CDCl_3_) δ** 7.35 (d, J = 8.7 Hz, 2H, 2*HAr), 6.86 (d, J = 8.8 Hz, 2H, 2*HAr), 5.46 (s, 1H, CHPMB), 5.17 (t, J = 10.0 Hz, 1H, H3), 4.72 (d, J = 7.8 Hz, 1H, H1), 4.26 (dd, J = 10.6, 5.1 Hz, 1H, H5), 4.07 (dd, J = 17.9, 9.3 Hz, 1H, H2), 3.83 – 3.76 (m, 4H, OMePMB, H6a), 3.70 (t, J = 9.4 Hz, 1H, H4), 3.53 – 3.43 (m, 1H, H6b), 2.39 – 2.22 (m, 2H, CH2 α Amide), 2.16 – 2.02 (m, 2H, CH2 α Ester), 1.74 – 1.49 (m, 4H, 2*CH2β), 1.25 (s, 44H, 22*CH2), 0.96 – 0.78 (m, 15H, tBu + 2*CH3), 0.09 (s, 3H, Si-Mea), 0.07 (s, 3H, Si-Meb).**^13^C NMR (400 MHz, CDCl_3_) δ** 175.33, 174.42, 159.17, 129.95, 128.75, 113.53, 102.03, 97.73, 78.46, 70.96, 67.91, 64.34, 56.01, 54.65, 37.75, 34.12, 31.20, 29.20 – 28.55, 25.33, 25.13, 22.84, 18.56, 14.01, -2.89, -3.29.

*(4aR,6S,7R,8R,8aS)-6-((tert-butyldimethylsilyl)oxy)-2-(4-methoxyphenyl)-7-oleamido-hexa*

*hydropyrano[3,2-d][1,3]dioxin-8-yl oleate (****4****):* **^1^H NMR (400 MHz, CDCl_3_) δ** 7.35 (d, J = 8.6 Hz, 2H, 2*HAr), 6.85 (d, J = 8.7 Hz, 2H, 2*HAr), 5.67 (d, J = 9.6 Hz, 1H, NH), 5.45 (s, 1H, H1 α), 5.40 – 5.23 (m, J = 10.2, 4.7 Hz, 4H, 4*CH=), 5.18 (t, J = 10.0 Hz, 1H, H3), 4.69 (d, J = 7.9 Hz, 1H,H1 β), 4.23 (dd, J = 10.5, 4.8 Hz, 1H, H5), 4.08 (dd, J = 18.2, 9.9 Hz, 1H, H2), 3.82 – 3.74 (m, 4H, MePMB, H6a), 3.69 (t, J = 9.5 Hz, 1H, H4), 3.53 – 3.39 (m, 1H, H6b), 2.40 – 2.22 (m, 2H, CH2 α Ester), 2.15 – 2.02 (m, 2H, , CH2 α Amide), 2.02 – 1.93 (m, 8H, 4*CH2-=), 1.62 – 1.46 (m, 4H, 4*CH2 β), 1.39 – 1.15 (m, 40H, 40*CH2), 0.94 – 0.77 (m, 15H, tBu, 2*Me), 0.07 (s, 3H, Si-Mea), 0.04 (s, 3H, Si-Meb). **^13^C NMR (400 MHz, CDCl_3_) δ** 174.28, 172.62, 160.02, 129.97, 129.66, 127.38, 113.49, 101.27, 97.33, 78.66, 71.72, 68.60, 66.58, 56.27, 55.22, 36.90, 34.27, 31.90, 29.77, 29.53, 29.37, 29.32, 29.10, 27.22, 25.56, 25.52, 25.02, 22.68, 17.82, 14.12, -4.12, -5.22.

*(9Z,12Z)-(4aR,6S,7R,8R,8aS)-6-((tert-butyldimethylsilyl)oxy)-2-(4-methoxyphenyl)-7-((9Z,12Z)-octadeca-9,12-dienamido)hexahydropyrano[3,2-d][1,3]dioxin-8-yl octadeca-9,12-dienoate (****5****)****:* ^1^H NMR (400 MHz, CDCl_3_) δ** 7.35 (d, J = 8.8 Hz, 2H, 2*CHAr), 6.86 (d, J = 8.8 Hz, 2H, 2*CHAr), 5.46 (s, 1H, H1 α), 5.36 (m, J = 16.6, 14.2, 8.0 Hz, 9H, 8*CH=, CHPMB), 5.16 (t, J = 10.0 Hz, 1H, H3), 4.73 (d, J = 7.9 Hz, 1H, H1 β), 4.27 (dd, J = 10.6, 5.1 Hz, 1H, H5), 4.06 (dd, J = 18.3, 9.7 Hz, 1H, H2), 3.79 (m, 4H, OMe, H6a), 3.70 (t, J = 9.4 Hz, 1H, H4), 3.47 (m, 1H, H6b), 2.76 (d, J = 3.8 Hz, 4H, 2*=CH2=), 2.30 (m, 2H, CH2 α Amide), 2.05 (m, 10H, 4*CH2-=, CH2 α Ester), 1.56 (m, 2H, 2*CH2 β), 1.29 (m, 28H, 14*CH2), 0.88 (m, 16H, tBu, 2*Me), 0.10 (s, 3H, Si-Mea), 0.08 (s, 3H, Si-Meb). **^13^C NMR (400 MHz, CDCl_3_) δ** 174.15, 172.61, 160.06, 130.21, 130.00, 127.87, 127.43, 113.52, 101.32, 97.41, 78.62, 71.47, 68.61, 66.70, 56.44, 55.25, 36.91, 34.26, 31.51, 29.51, 27.19, 25.61, 25.52, 25.00, 22.57, 17.85, 14.09, -4.09, -5.27.

*(4aR,6S,7R,8R,8aS)-7-azido-6-((tert-butyldimethylsilyl)oxy)-2-(4-methoxyphenyl) hexahydro*

*pyrano[3,2-d][1,3]dioxin-8-yl tetradecanoate (****6****):* **^1^H NMR (400 MHz, CDCl_3_) δ** 7.33 (d, J = 8.7 Hz, 2H, 2*CHAr), 6.85 (d, J = 8.7 Hz, 2H, 2*CHAr), 5.43 (s, 1H, CH-PMP), 5.12 (t, J = 9.9 Hz, 1H, H3), 4.71 (d, J = 7.6 Hz, 1H, H1), 4.27 (dd, J = 10.4, 4.8 Hz, 1H, H5), 3.82 – 3.72 (m, 4H, OMe+H6a), 3.61 (t, J = 9.5 Hz, 1H, H4), 3.52 – 3.44 (m, 1H, H6b), 3.44 – 3.34 (m, 1H, H2), 2.36 (t, J = 7.4 Hz, 2H, CH2α), 1.70 – 1.54 (m, 4H, CH2β), 1.36 – 1.14 (m, 20H, 10*CH2), 0.94 (s, 9H,tBu), 0.88 (t, J = 6.8 Hz, 3H, Me), 0.23 – 0.13 (m, 6H, 2*Me-Si). **^13^C NMR (400 MHz, CDCl_3_) δ** 174.42, 159.22, 129.95, 128.73, 113.58, 102.03, 100.29, 78.80, 71.37, 67.93, 64.37, 56.08, 46.54, 34.14, 31.67, 29.12 – 28.74, 25.76 – 25.67, 25.33, 22.94, 18.57, 14.01, -2.89, -3.29.

*(4aR,6S,7R,8R,8aS)-7-amino-6-((tert-butyldimethylsilyl)oxy)-2-(4-methoxyphenyl) hexahydro*

*pyrano[3,2-d][1,3]dioxin-8-yl tetradecanoate (****7****):* **^1^H NMR (400 MHz, CDCl_3_) δ** 7.34 (d, J = 8.6 Hz, 2H, 2*CHAr), 6.84 (d, J = 8.6 Hz, 2H, 2*CHAr), 5.43 (s, 1H,CH-PMP), 5.10 (t, J = 9.8 Hz, 1H, H3), 4.57 (d, J = 7.6 Hz, 1H, H1), 4.25 (dd, J = 10.4, 4.8 Hz, 1H, H5), 3.82 – 3.67 (m, 4H, H6a +OMe), 3.63 (t, J = 9.4 Hz, 1H, H4), 3.49 (td, J = 9.7, 5.0 Hz, 1H, H6b), 2.82 (dd, J = 9.8, 7.6 Hz, 1H, H2), 2.35 (t, J = 7.4 Hz, 2H, CH2α), 1.67 – 1.53 (m, 2H, CH2β), 1.48 (s, 2H, NH_2_), 1.17 (m, 20H, 10*CH_2_), 0.91 (s, 9H, tBu), 0.87 (t, J = 6.7 Hz, 3H, Me), 0.13 (s, 6H, 2*Si-Me).**^13^C NMR (400 MHz, CDCl_3_) δ** 174.45, 159.20, 129.98, 77, 113.58, 102.04, 99.65, 77.65, 71.69, 67.93, 64.38, 58.11, 56.04, 34.15, 31.65, 29.36 – 28.85 (m), 25.66 – 25.55, 25.33, 22.94, 18.59, 14.02, -2.89 – -3.29.

*(4aR,6S,7R,8R,8aS)-6-((tert-butyldimethylsilyl)oxy)-2-(4-methoxyphenyl)-7-((9Z,12Z)-octa*

*deca-9,12-dienamido)hexahydropyrano[3,2-d][1,3]dioxin-8-yl tetradecanoate (****8****):* **^1^H NMR (400 MHz, CDCl_3_) δ** 7.35 (d, J = 8.7 Hz, 2H, 2*CHAr), 6.86 (d, J = 8.8 Hz, 2H, 2*CHAr), 5.46 (s, 1H, CHPMB), 5.42 – 5.26 (m, 5H, 4*CH= +H1β), 5.19 (t, J = 10.0 Hz, 1H, H3), 4.72 (d, J = 7.9 Hz, 1H, H1α), 4.26 (dd, J = 10.5, 5.0 Hz, 1H, H5), 4.11 – 4.03 (m, 1H, H2), 3.80 – 3.66 (m, 5H, OMe+H6a+H4), 3.44 (dd, J = 9.6, 4.9 Hz, 1H, H6b), 2.76 (t, J = 6.5 Hz, 2H, =CH2=), 2.39 – 2.23 (m, 2H, CH2α Amide), 2.16 – 1.99 (m, 6H, 2*CH2= + CH2α Ester), 1.64 – 1.48 (m, 4H, 2*CH2β), 1.40 – 1.14 (m, 34H, 17*CH2), 0.91 – 0.81 (m, 15H, tBu+2*Me), 0.08 (s, 3H, Si-Mea), 0.06 (s, 3H, Si-Meb).**^13^C NMR (400 MHz, CDCl_3_) δ** 175.05, 174.33, 172.65, 160.04, 130.21, 128.04, 113.53, 101.35, 96.40, 78.66, 69.95, 68.62, 66.55, 62.71, 55.58, 55.23, 36.89, 34.29, 31.92, 31.51, 30.39 – 28.18, 27.18, 25.61, 22.69, 22.57, 17.89, 14.12, -4.04, -5.21.

*(4aR,6S,7R,8R,8aS)-6-((tert-butyldimethylsilyl)oxy)-2-(4-methoxyphenyl)-7-oleamidohexa*

*hydropyrano[3,2-d][1,3]dioxin-8-yl tetradecanoate (****9****):* **^1^H NMR (400 MHz, CDCl_3_) δ** 7.35 (d, J = 8.7 Hz, 2H, 2CHAr), 6.86 (d, J = 8.8 Hz, 2H, 2CHAr), 5.50 – 5.40 (m, 2H, H1a, CHPMB), 5.34 (t, J = 6.1 Hz, 2H, 2*CH=), 5.17 (t, J = 10.1 Hz, 1H, H3), 4.73 (d, J = 7.9 Hz, 2H,H1b), 4.27 (dd, J = 10.5, 4.8 Hz, 1H, H5), 4.06 (dd, J = 18.3, 9.6 Hz, 1H, H2), 3.83 – 3.74 (m, 4H, OMe+H6a), 3.70 (t, J = 9.4 Hz, 1H, H4), 3.55 – 3.41 (m, 1H, H6b), 2.40 – 2.20 (m, 2H, CH2a Amide), 2.16 – 2.04 (m, 2H, CH2a Ester), 2.00 (d, J = 5.9 Hz, 4H, 2*CH2-=), 1.55 (s, 4H, 2*CH2b), 1.39 – 1.13 (m, 40H, 20*CH2), 0.95 – 0.83 (m, 15H, tBu+2*Me), 0.09 (s, 3H, Mea-Si), 0.07 (s, 3H, Meb-Si). **^13^C NMR (400 MHz, CDCl_3_) δ** 174.30, 172.62, 160.02, 129.97, 129.67, 127.37, 114.29, 113.51, 101.25, 97.34, 78.66, 71.69, 68.60, 66.57, 56.26, 55.22, 36.91, 34.28, 31.92, 29.43, 27.22, 25.51, 25.03, 22.68, 17.82, 14.12, -4.01, -5.17.

*(2R,3R,4R,5S,6R)-2,5-dihydroxy-6-(((4-methoxybenzyl)oxy)methyl)-3-tetradecanamidotetra*

*hydro-2H-pyran-4-yl tetradecanoate (****3a****):* **^1^H NMR (400 MHz, CDCl_3_) δ** 7.25 (d, J = 9.4 Hz, 2H, 2*CHAr), 6.87 (d, J = 8.6 Hz, 2H, 2*CHAr), 5.89 (d, J = 9.1 Hz, 1H, H1a), 5.22 – 5.09 (m, 2H, H1b +H3), 4.50 (m, 2H, CH2 PMB), 4.25 – 4.14 (m, 1H, H2), 4.03 (m, 1H, H5), 3.79 (s, 3H, OMe), 3.76 – 3.59 (m, 3H, H4+ 2*H6), 2.33 (m, 2H, 2*CH2a Amide), 2.24 – 2.02 (m, 2H, 2*CH2a Ester), 1.63 – 1.46 (m, 4H, 2*CH2b), 1.37 – 1.16 (m, 20H, 10*CH2), 0.87 (t, J = 6.7 Hz, 6H, 2*Me).**^13^C NMR (400 MHz, CDCl_3_) δ** 175.14, 173.33, 159.54, 129.51, 113.73, 91.91, 73.25, 70.42, 69.86, 69.39, 55.30, 36.88, 34.35, 31.91, 30.42 - 28.37, 25.62, 23.79, 24.74, 22.78, 14.10.

*(2R,3R,4R,5S,6R)-2,5-dihydroxy-6-(((4-methoxybenzyl)oxy)methyl)-3-oleamidotetrahydro-2H-pyran-4-yl oleate (****4a****):* **^1^H NMR (400 MHz, CDCl_3_) δ** 7.26 (s, 2H, 2*CHAr), 6.88 (d, J = 8.6 Hz, 2H, 2*CHAr), 5.77 (d, J = 9.4 Hz, 1H, H1), 5.34 (s, 4H, 4*CH=), 5.19 – 5.10 (m, 1H, H3), 4.52 (q, J = 11.6 Hz, 2H, CH2 PMB), 4.29 – 4.15 (m, 1H, H2), 4.10 – 3.97 (m, 1H, H5), 3.80 (s, 3H, MePMB), 3.74 – 3.63 (m, J = 8.1 Hz, 3H, H4+2*H6), 2.33 (dd, J = 13.8, 7.4 Hz, 2H, CH2 α amide), 2.12 (dd, J = 12.9, 7.5 Hz, 2H, CH2 α ester), 2.00 (d, J = 5.9 Hz, 8H,4*CH2-CH=), 1.57 (s, 8H, 2*CH2 β), 1.26 (s, 45H, 20*CH2), 0.88 (t, J = 6.8 Hz, 6H, 2*Me). **^13^C NMR (400 MHz, CDCl_3_) δ** 175.13, 173.15, 159.35 130.01, 129.68, 129.53, 113.83, 91.84, 73.36, 73.25, 70.49, 70.01, 69.85, 55.27, 51.72, 36.71, 34.31, 31.91, 30.33 - 28.11, 27.20, 25.62, 24.93, 22.69, 14.15.

*(9Z,12Z)-(2R,3R,4R,5S,6R)-2,5-dihydroxy-6-(((4-methoxybenzyl)oxy)methyl)-3-((9Z,12Z)-octadeca-9,12-dienamido)tetrahydro-2H-pyran-4-yl octadeca-9,12-dienoate (****5a****):* **^1^H NMR (400 MHz, CDCl_3_) δ** 7.29 – 7.19 (m, 2H, 2*CHAr), 6.88 (d, J = 8.6 Hz, 2H, 2*CHAr), 5.76 (d, J = 9.7 Hz, 1H, H1β), 5.44 – 5.25 (m, 8H, 8*CH=), 5.23 (s, 1H, H1α), 5.20 – 5.09 (m, 1H, H3), 4.59 – 4.43 (m, 2H, CH2 PMB), 4.22 (td, J = 11.0, 3.7 Hz, 1H, H2), 4.06 – 4.01 (m, 1H, H5), 3.80 (s, 3H, OMe), 3.69 (d, J = 4.8 Hz, 3H, 2*H6+H4), 2.76 (t, J = 6.2 Hz, 4H, 2*=CH2=), 2.40 – 2.28 (m, 2H, CH2 α amide), 2.16 – 2.08 (m, 2H, CH2 α ester), 2.07 – 1.95 (m, 8H, 4*CH2-=), 1.73 – 1.48 (m, 4H, 2*CH2β), 1.40 – 1.18 (m, 28H, 14*CH2), 0.88 (t, J = 6.8 Hz, 6H, 2*Me). **^13^C NMR (400 MHz, CD_3_OD) δ** 171.12, 169.14, 155.38, 126.27, 126.03, 125.65, 125.54, 124.08, 123.92, 109.86, 87.85, 69.38, 66.48, 66.06, 65.91, 64.89, 51.30, 47.84, 32.76, 30.36, 27.56, 26.24 – 24.74, 23.24, 21.66, 18.63, 17.11, 10.24.

*(2R,3R,4R,5S,6R)-2,5-dihydroxy-6-(((4-methoxybenzyl)oxy)methyl)-3-((9Z,12Z)-octadeca-9,12-dienamido)tetrahydro-2H-pyran-4-yl tetradecanoate (****8a****):* **^1^H NMR (400 MHz, CDCl_3_) δ** 7.27 (d, J = 8.6 Hz, 2H, 2*CHAr), 6.88 (d, J = 8.6 Hz, 2H, 2*CHAr), 5.94 (d, J = 9.2 Hz, 1H, H1β), 5.43 – 5.26 (m, 4H, 4*CH=), 5.21 (d, J = 2.3 Hz, 1H, H1α), 5.17 – 5.10 (m, 1H, H3), 4.58 – 4.43 (m, 2H, CH2 PMB), 4.23 – 4.12 (m, 1H, H2), 4.10 – 4.01 (m, 1H, H5), 3.80 (s, 3H, OMe), 3.75 – 3.66 (m, 3H, H4+2*H6), 2.76 (t, J = 5.9 Hz, 2H, =CH2=), 2.43 – 2.30 (m, 2H, CH2α Amide), 2.16 – 2.07 (m, 2H, CH2α Ester), 2.08 – 1.98 (m, 4H, 2*CH2-=), 1.69 – 1.49 (m, 4H, 2*CH2β), 1.44 – 1.19 (m, 34H, 17*CH2), 0.94 – 0.82 (m, 6H, 2*Me). **^13^C NMR (400 MHz, CDCl_3_) δ** 175.25, 173.51, 159.79, 130.10, 129.66, 128.04, 127.86, 113.90, 91.71, 74.70, 73.38, 70.02, 67.97, 59.95, 55.29, 51.84, 40.79, 36.73, 34.34, 29.92, 27.18, 25.61, 24.94, 22.63, 14.13.

*(2R,3R,4R,5S,6R)-2,5-dihydroxy-6-(((4-methoxybenzyl)oxy)methyl)-3-oleamidotetrahydro-2H-pyran-4-yl tetradecanoate (****9a****):* **^1^H NMR (400 MHz, CDCl_3_) δ** 7.29 – 7.21 (m, 2H, 2*CHAr), 6.88 (d, J = 8.5 Hz, 2H, 2*CHAr), 5.91 (d, J = 9.3 Hz, 1H, H1α), 5.41 – 5.28 (m, 2H, 2*CH=), 5.20 (s, 1H, H1β), 5.19 – 5.09 (m, 1H, H3), 4.51 (q, J = 11.5 Hz, 2H, CH2 PMB), 4.23 – 4.13 (m, 1H, H2), 4.07 – 3.98 (m, 1H, H5), 3.80 (s, 3H, OMe), 3.74 – 3.64 (m, 3H, 2*H6+H4), 2.42 – 2.27 (m, 2H, CH2a Amide), 2.20 – 2.06 (m, 2H, CH2a Ester), 2.06 – 1.91 (m, 4H, 2*CH2-=), 1.64 – 1.48 (m, 4H, 2*CH2β), 1.39 – 1.17 (m, 40H, 20*CH2), 0.87 (t, J = 6.7 Hz, 6H, 2*Me). **^13^C NMR (400 MHz, CD_3_OD) δ** 175.26, 173.21, 163.34, 130.16, 130.16, 129.85, 129.78, 129.65, 114.00, 92.08, 73.55, 72.14, 70.72, 70.16, 69.24, 55.43, 51.76, 36.91, 34.48, 32.08, 30.54 – 28.46, 27.38, 27.33, 25.76, 25.11, 22.85, 14.29.

*4,4'-(((2S,3R,4R,5S,6R)-6-(((4-methoxybenzyl)oxy)methyl)-3-tetradecanamido-4-(tetra*

*decaneoyloxy)tetrahydro-2H-pyran-2,5-diyl)bis(oxy))bis(4-oxobutanoic acid) (****3b****):* **^1^H NMR (400 MHz, CD_3_OD) δ** 7.25 (d, J = 8.4 Hz, 2H, 2*CHa Ar), 6.88 (d, J = 8.4 Hz, 2H, 2*CHb Ar), 6.11 (d, J = 3.4 Hz, 1H, H1), 5.35 – 5.26 (m, 1H, H3), 5.22 (t, J = 9.7 Hz, 1H, H4), 4.46 – 4.33 (m, 3H, H2, CH2 PMB), 4.08 – 4.00 (m, 1H, H5), 3.78 (s, 3H, OMe), 3.67 – 3.50 (m, 2H, 2*H6), 2.78 – 2.41 (m, 8H, 4*CH2-COO), 2.37 – 2.21 (m, 4H, 2*CH2α), 1.62 – 1.48 (m, 4H,2*CH2β), 1.39 – 1.21 (m, 40H, 20*CH2), 0.97 – 0.82 (m, 6H, 2*Me). **^13^C NMR (400 MHz, CD_3_OD) δ** 174.92, 174.54, 173.11, 171.03, 170.90, 147.45, 129.45, 129.30, 113.01, 90.26, 74.25, 72.52, 70.61, 70.00, 60.83, 53.99, 50.52, 33.34, 31.45, 30.33 – 27.03, 24.30, 22.11, 12.82.

*4,4'-(((2S,3R,4R,5S,6R)-6-(((4-methoxybenzyl)oxy)methyl)-3-oleamido-4-(oleoyloxy)*

*tetrahydro-2H-pyran-2,5-diyl)bis(oxy))bis(4-oxobutanoic acid) (****4b****):* **^1^H NMR (400 MHz, CDCl_3_) δ** 7.24 (d, J = 8.6 Hz, 2H, 2*CHAr), 6.86 (d, J = 8.6 Hz, 2H, 2*CHAr), 6.21 (d, J = 3.3 Hz, 1H, H1α), 5.87 (d, J = 8.3 Hz, 1H, H1β), 5.47 – 5.29 (m, 4H, 4*CH=), 5.26 (t, J = 10.0 Hz, 1H, H4), 5.20 – 5.09 (m, 1H, H3), 4.54 – 4.33 (m, 3H, CH2PMB, H2), 3.88 (d, J = 9.2 Hz, 1H, H5), 3.79 (s, 3H, OMe), 3.58 – 3.42 (m, 2H, 2*H6), 2.90 – 2.36 (m, 8H, 4*CH2-COO), 2.26 (t, J = 7.5 Hz, 2H, CH2α ester), 2.08 (dd, J = 13.2, 7.0 Hz, 2H, CH2α amide), 2.00 (d, J = 5.3 Hz, 8H, 4*CH2-=), 1.53 (s, 4H, 2*CH2β), 1.26 (s, 40H, 20*CH2), 0.87 (t, J = 6.6 Hz, 6H, 2*Me). **^13^C NMR (400 MHz, CDCl_3_) δ** 172.12, 169.73, 168.12, 164.28, 163.88, 154.05, 124.86, 124.81, 124.57, 124.49, 124.43, 124.37, 108.50, 86.13, 69.83, 67.99, 65.97, 63.33, 62.48, 50.08, 45.83, 31.19 - 28.96, 26.72, 24.03, 22.02, 20.26, 19.62, 17.51, 8.96.

*4,4'-(((2S,3R,4R,5S,6R)-6-(((4-methoxybenzyl)oxy)methyl)-3-((9Z,12Z)-octadeca-9,12-dien*

*amido)-4-((9Z,12Z)-octadeca-9,12-dienoyloxy)tetrahydro-2H-pyran-2,5-diyl)bis(oxy))bis(4-oxobutanoic acid) (****5b****):* **^1^H NMR (400 MHz, CDCl_3_) δ** 7.28 – 7.19 (m, 2H, 2*CHAr), 6.86 (d, J = 8.6 Hz, 2H, 2*CHAr), 6.21 (d, J = 3.4 Hz, 1H, H1a), 5.45 – 5.28 (m, 8H, 8*CH=), 5.25 (t, J = 8.6 Hz, 1H, H4), 5.14 (t, J = 10.6 Hz, 1H, H3), 4.53 – 4.34 (m, 3H, H2+ CH2 PMB), 3.93 – 3.82 (m, 1H, H5), 3.80 (s, 3H,OMe), 3.58 – 3.42 (m, 2H, 2*H6), 2.87 – 2.72 (m, 4H, 2*=CH2=), 2.72 – 2.52 (m, 8H, 4*CH2-COO), 2.44 – 2.38 (m, 2H, CH2 a Amide), 2.28 (t, J = 7.7 Hz, 2H, CH2 a Ester), 2.13 – 1.97 (m, 8H, 4*CH2=), 1.64 – 1.46 (m, 4H, 2*CH2 b), 1.42 – 1.17 (m, 28H, 14*CH2), 0.88 (t, J = 6.8 Hz, 6H, 2*Me).**^13^C NMR (101 MHz, CDCl_3_) δ** 173.72, 172.99, 169.41, 168.69, 130.26, 129.74, 128.10, 127.83, 113.88, 90.88, 73.10, 70.89, 67.65, 65.78, 55.27, 53.78, 31.51, 30.75 – 28.09, 27.20, 25.61, 24.75, 22.57, 14.09.

*4,4'-(((2S,3R,4R,5S,6R)-6-(((4-methoxybenzyl)oxy)methyl)-3-((9Z,12Z)-octadeca-9,12-dien*

*amido)-4-(tetradecanoyloxy)tetrahydro-2H-pyran-2,5-diyl)bis(oxy))bis(4-oxobutanoic acid) (****8b****):* **^1^H NMR (400 MHz, CDCl_3_) δ** 7.23 (d, J = 8.9 Hz, 2H, 2*CHAr), 6.85 (d, J = 8.3 Hz, 2H, 2*CHAr), 6.21 (d, J = 2.8 Hz, 1H, H1), 5.45 – 5.33 (m, 4H, 4*CH=), 5.19 -5.14 (m, 2H, H4 + H3), 4.53 – 4.35 (m, 2H, CH2 PMB), 4.30 – 4.21 (m, 1H, H2), 4.00 - 3.98 (m, 1H, H5), 3.79 (s, 3H, OMe), 3.57 – 3.41 (m, 2H, 2*H6), 2.81 – 2.42 (m, 10H, 4*CH2-COO + =CH2=), 2.40 – 2.14 (m, 4H, 2*CH2a), 2.09 - 2.00 (m, 4H, 2*CH2=), 1.57 -1.50 (m, 4H, 2*CH2b), 1.34 - 1.32 (m, 34H, 17*CH2), 1.07 – 0.75 (m, 3H, 2*Me).**^13^C NMR (400 MHz, CDCl_3_) δ** 175.72, 175.36, 174.47, 172.98, 171.81, 159.06, 132.53, 130.17, 129.07, 128.52, 113.44, 91.45, 73.64, 71.52, 70.66, 69.21, 56.03, 54.41, 37.80, 34.14, 31.18, 30.10, 29.34 - 27.91, 25.33, 25.12, 23.01, 14.08.

*4,4'-(((2S,3R,4R,5S,6R)-6-(((4-methoxybenzyl)oxy)methyl)-3-oleamido-4-(tetradecane*

*oyloxy)tetrahydro-2H-pyran-2,5-diyl)bis(oxy))bis(4-oxobutanoic acid) (****9b****):* **^1^H NMR (400 MHz, CDCl_3_) δ** 7.23 (d, J = 8.6 Hz, 2H, 2*CHAr), 6.86 (d, J = 8.7 Hz, 2H, 2*CHAr), 6.22 (d, J = 2.9 Hz, 1H, H1b), 5.43 – 5.28 (m, 2H, 2*CH=), 5.23 (t, J = 9.1 Hz, 1H, H4), 5.16 (t, J = 9.9 Hz, 1H, H3), 4.55 – 4.34 (m, 2H, CH2 PMB), 4.21 – 4.11 (m, 1H, H2), 3.94 – 3.87 (m, 1H, H5), 3.80 (s, 3H, OMe), 3.58 – 3.43 (m, 2H, 2*H6), 2.75 – 2.56 (m, 8H, 4*CH2-COO), 2.26 (t, J = 7.7 Hz, 2H, CH2a Amide), 2.08 (t, J = 7.3 Hz, 2H, , CH2a Ester), 2.03 – 1.90 (m, 4H, 2*CH2-=), 1.60 – 1.46 (m, 4H, , CH2b), 1.41 – 1.14 (m, 40H, 20*CH2), 0.87 (t, J = 6.4 Hz, 6H, 2*Me).**^13^C NMR (400 MHz, CDCl_3_) δ** 177.79, 176.65, 173.34, 172.62, 168.94, 159.27, 129.98, 129.63, 113.71, 91.22, 73.24, 71.20, 68.69, 67.81, 55.27, 51.91, 36.32, 34.34, 31.92, 29.94 - 28.37, 27.19, 25.40, 24.68, 22.77, 14.05.

*4,4'-(((2S,3R,4R,5S,6R)-6-(hydroxymethyl)-3-oleamido-4-(oleoyloxy)tetrahydro-2H-pyran-2,5-diyl)bis(oxy))bis(4-oxobutanoic acid) (****FP13****):* **^1^H NMR (400 MHz, CD_3_OD) δ** 6.15 (d, J = 3.2 Hz, 1H, H1), 5.41 – 5.28 (m, 5H, 4*CH=, H3), 5.16 (t, J = 9.9 Hz, 1H, H4), 4.41 (dd, J = 10.5, 3.4 Hz, 1H, H2), 3.96 (ddd, J = 7.5, 4.7, 2.3 Hz, 1H, H5), 3.69 – 3.52 (m, 2H, 2*H6), 2.80 – 2.48 (m, 8H, 4*CH2-COO), 2.40 – 2.21 (m, 2H, CH2 α amide), 2.17 (t, J = 5.1 Hz, 2H, CH2 α ester), 2.11 – 1.95 (m, 4H, 4*CH2-=), 1.69 – 1.47 (m, 4H, 2*CH2 β), 1.46 – 1.16 (m, 40H,20*CH2), 1.01 – 0.77 (m, 6H, 2*Me). **^13^C NMR (400 MHz, CD_3_OD) δ** 175.15, 174.79, 173.30, 171.45, 171.16, 129.41, 90.51, 72.22, 70.21, 68.49, 60.04, 50.59, 35.38, 33.58, 31.68 - 29.02, 26.76, 25.57, 24.55, 22.35, 13.08. **MS (ESI-)** m/z calcd (M-H) C50H84NO13-= 906,59, found = 906.60.

*4,4'-(((2S,3R,4R,5S,6R)-6-(hydroxymethyl)-3-((9Z,12Z)-octadeca-9,12-dienamido)-4-((9Z,12Z)-octadeca-9,12-dienoyloxy)tetrahydro-2H-pyran-2,5-diyl)bis(oxy))bis(4-oxobutanoic acid) (****FP14****):* **^1^H NMR (400 MHz, CDCl_3_) δ** 6.19 (d, J = 2.8 Hz, 1H, H1α), 5.90 (d, J = 7.8 Hz, 1H, H1β), 5.44 – 5.28 (m, 8H, 8*CH=), 5.28 – 5.21 (m, 1H, H3), 5.21 – 5.12 (m, 1H, H4), 4.44 (t, J = 9.0 Hz, 1H, H2), 3.87 – 3.75 (m, 1H, H5), 3.70 (s, 1H, H6a), 3.60 (s, 1H, H6b), 2.76 (t, J = 6.0 Hz, 4H, 2*=CH2=), 2.72 – 2.53 (m, 8H, 2*CH2-COOH), 2.40 – 2.24 (m, 4H, 2*CH2α), 2.10 – 1.99 (m, 8H,4*CH2-COOH), 1.61 – 1.45 (m, 4H, 2*CH2), 1.45 – 1.11 (m, 28H, 14*CH2), 0.96 – 0.78 (m, 6H, 2*Me). **^13^C NMR (400 MHz, CDCl_3_) δ** 176.78, 176.42, 174.81, 173.46, 170.72, 169.68, 130.24, 127.85, 91.24, 72.18, 70.16, 68.04, 60.88, 51.13, 36.41, 34.13, 31.84, 31.53 - 27.19, 25.53, 24.86, 22.63, 14.11. **MS (ESI-)** m/z calcd (M-H) C50H80NO13-= 902,56, found = 902.60

*4,4'-(((2S,3R,4R,5S,6R)-6-(hydroxymethyl)-3-oleamido-4-(tetradecanoyloxy)tetrahydro-2H-pyran-2,5-diyl)bis(oxy))bis(4-oxobutanoic acid) (****FP15****):* **^1^H NMR (400 MHz, CDCl_3_) δ** 6.20 (d, J = 3.3 Hz, 1H, H1 α), 5.90 (d, J = 8.7 Hz, 1H, H1 β), 5.40 – 5.27 (m, 2H, 2*CH=), 5.28 – 5.12 (m, 2H, H3+H4), 4.42 (t, J = 8.4 Hz, 1H, H2), 3.82 – 3.75 (m, 1H, H5), 3.74 – 3.53 (m, 2H, 2*H6), 2.85 – 2.54 (m, 8H, 4*CH2-COOH), 2.29 (t, J = 7.6 Hz, 2H, CH2 α Amide), 2.15 – 2.05 (m, 2H, CH2 α Ester), 2.05 – 1.94 (m, 4H, 2*CH2-=), 1.61 – 1.47 (m, 4H, 2*CH2 β), 1.29 (d, J = 28.5 Hz, 40H, 20*CH2), 0.88 (t, J = 6.8 Hz, 6H, 2*Me). **^13^C NMR (400 MHz, CDCl_3_) δ** 176.41, 176.15, 174.85, 173.99, 171.21, 170.29, 129.99, 129.64, 91.09, 72.19, 70.19, 68.20, 60.80, 51.08, 36.35, 34.14, 29.45 - 27.21, 27.18, 24.88, 14.12. **MS (ESI-)** m/z calcd (M-H) C46H78NO13- =852,55, found = 852.60

*4,4'-(((2S,3R,4R,5S,6R)-6-(hydroxymethyl)-3-((9Z,12Z)-octadeca-9,12-dienamido)-4-(tetradecaneoyloxy)tetrahydro-2H-pyran-2,5-diyl)bis(oxy))bis(4-oxobutanoic acid) (****FP16****):* **^1^H NMR (400 MHz, CDCl_3_) δ** 6.19 (s, 1H, H1α), 5.91 (d, J = 6.8 Hz, 1H, H1β), 5.43 – 5.30 (m, 4H, 4*CH=), 5.27 (dd, J = 23.7, 9.8 Hz, 1H, H3), 5.21 – 5.11 (m, 1H, H4), 4.50 – 4.37 (m, 1H, H2), 3.82 (dd, J = 9.5, 3.2 Hz, 1H, H5), 3.77 – 3.53 (m, 2H, H6a+H6b), 2.83 – 2.73 (m, 2H, 4*=CH2=), 2.73 – 2.49 (m, 8H, 2*CH2-COOH), 2.39 – 2.23 (m, 4H, 2*CH2α), 2.10 – 1.98 (m, 4H,2*CH2-=), 1.53 (dd, J = 5.4, 2.9 Hz, 4H, 2*CH2β), 1.43 – 1.09 (m, 34H, 17*CH2), 0.86 (m, 6H, 2*Me). **^13^C NMR (400 MHz, CDCl_3_) δ** 176.57, 176.33, 174.83, 173.44, 171.65, 170.71, 130.24, 127.85, 91.23, 72.16, 70.12, 68.15, 60.82, 51.19, 36.41, 34.00, 31.92, 31.51, 30.82 – 27.86, 27.07, 25.61, 24.89, 22.69, 14.13. **MS (ESI-)** m/z calcd (M-H) C46H76NO13- = 850,53, found = 850.50

*4,4'-(((2S,3R,4R,5S,6R)-6-(hydroxymethyl)-3-tetradecanamido-4-(tetradecane*

*oyloxy)tetrahydro-2H-pyran-2,5-diyl)bis(oxy))bis(4-oxobutanoic acid) (****FP17****):* **^1^H NMR (400 MHz, CDCl_3_) δ** 6.19 (s, 1H, H1a), 5.94 (d, J = 7.4 Hz, 1H, H1b), 5.32 – 5.20 (m, 1H, H3), 5.20 – 5.07 (m, 1H, H4), 4.51 – 4.37 (m, 1H, H2), 3.89 – 3.75 (m, 1H, H5), 3.75 – 3.52 (m, 2H, 2*H6), 2.85 – 2.48 (m, 8H, 4*CH2-COO), 2.39 – 2.22 (m, 2H, CH2 Amide), 2.16 – 1.96 (m, 2H, CH2 Ester), 1.64 – 1.45 (m, 4H, 2*CH2b), 1.44 – 1.00 (m, 40H, 20*CH2), 0.99 – 0.80 (m, 6H, 2*Me). **^13^C NMR (400 MHz, CD_3_OD) δ** 176.30, 174.97, 173.70, 170.96, 170.01, 91.31, 72.30, 70.28, 68.31, 61.00, 55.05, 34.26, 32.03 - 29.43, 25.59, 25.00, 22.81, 14.26. **MS (ESI-)** m/z calcd (M-H) C42H72NO13-= 798,50, found = 798.50.


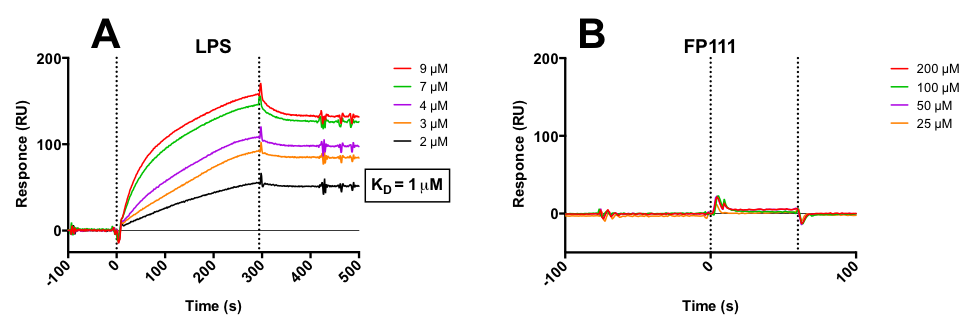


Figure S7**.** SPR binding studies on purified hMD-2 receptor. (A) SPR analysis show direct interaction between LPS and hMD-2 (K_D_ value is reported). Results are representative of at least three independent experiments.

| 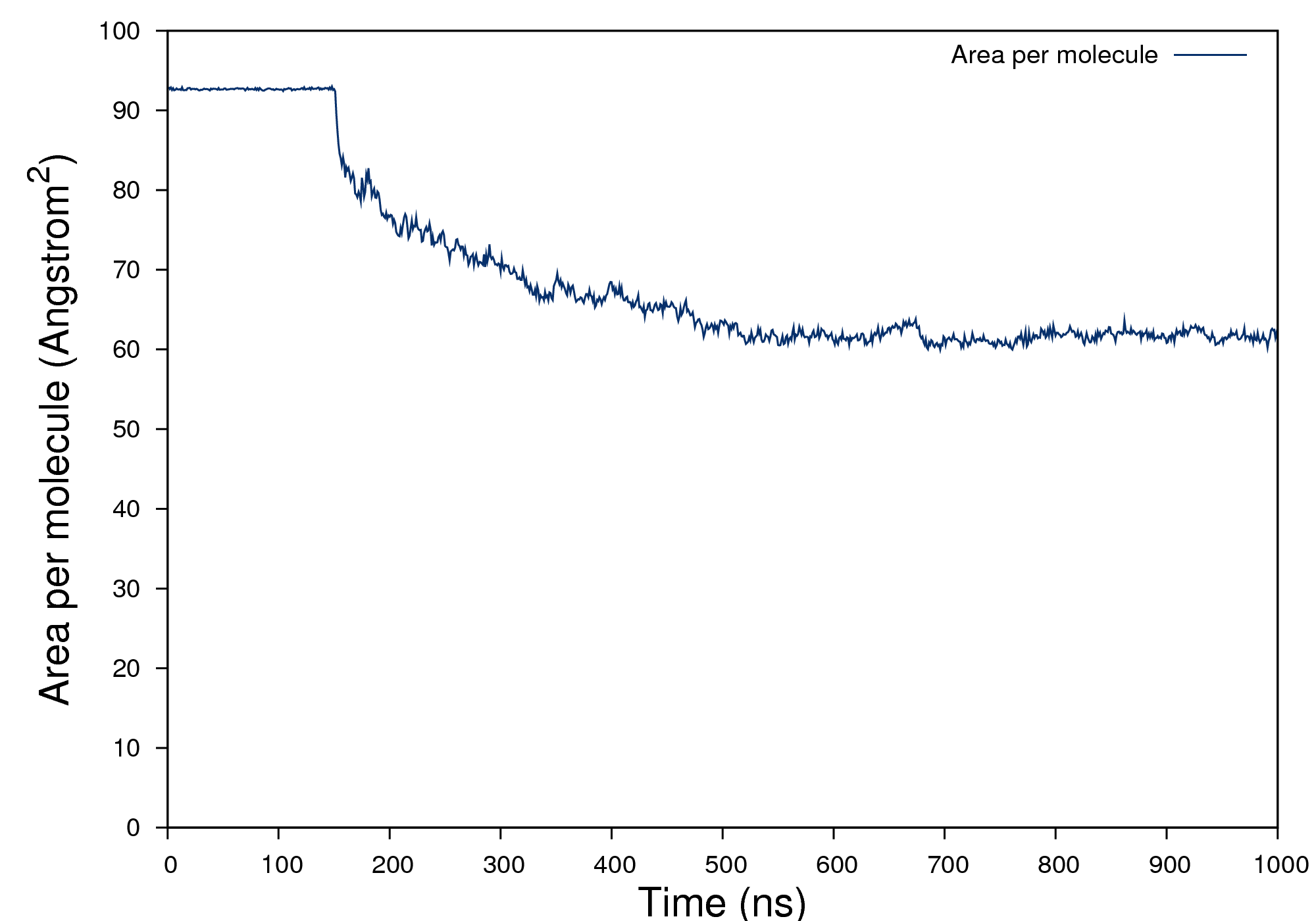 | 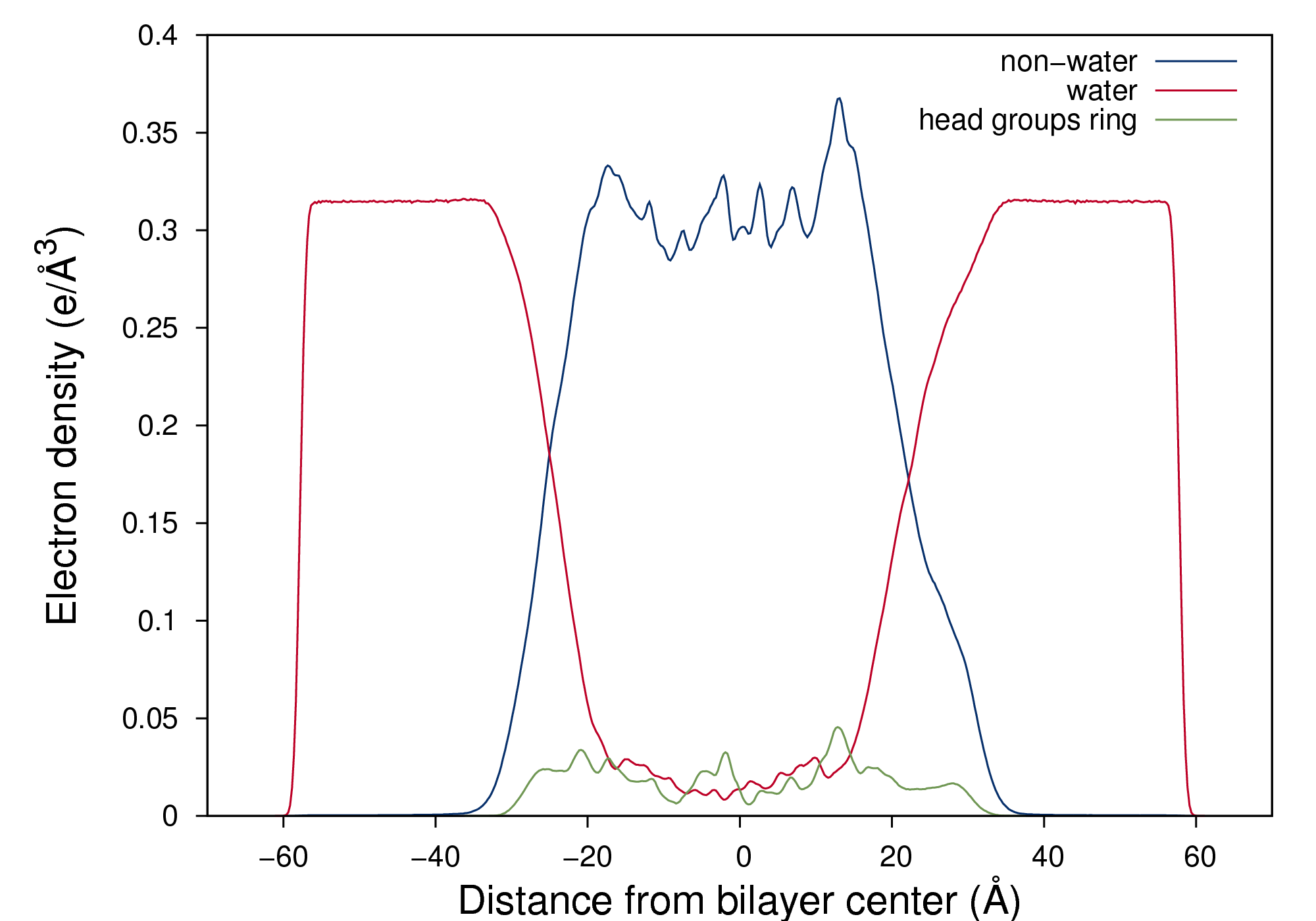 |
| --- | --- |

Figure S8. One the left: area per molecule over time. At t=150ns the system was switched from an isotropic to anisotropic pressure scaling. On the right: decomposed electron density of the system averaged over the 300 last ns of simulation.


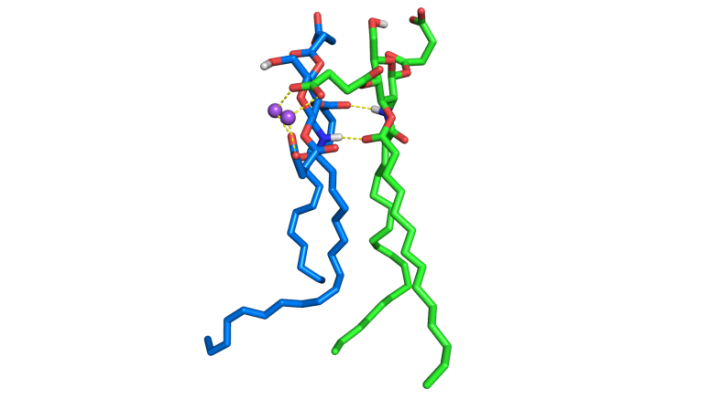

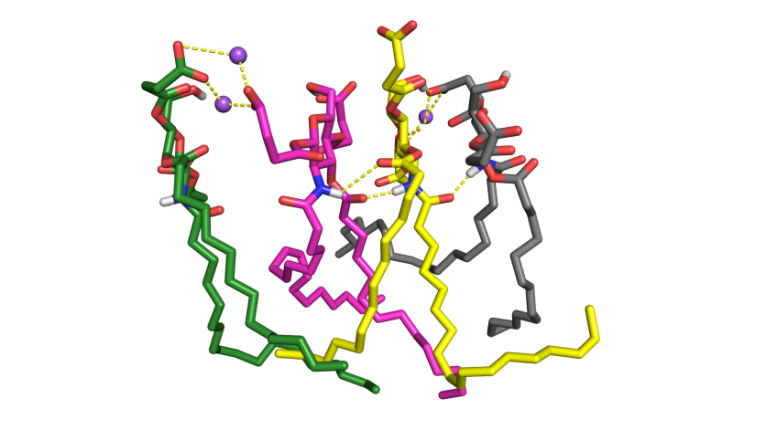

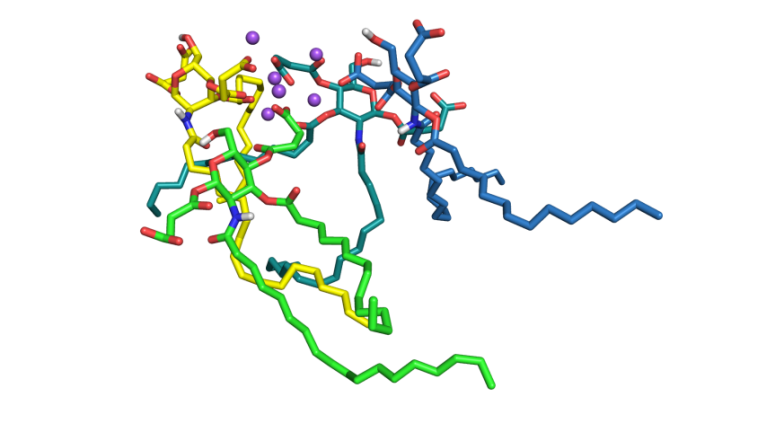


Figure S9**.** On the left: face to face packaging of two **FP15**. In the middle: parallel packaging of four **FP15**. On the right: clustering of four **FP15** around a Na^+^ rich pocket. Each **FP15** unit has a different color to help to visually differentiate individual molecule.

**
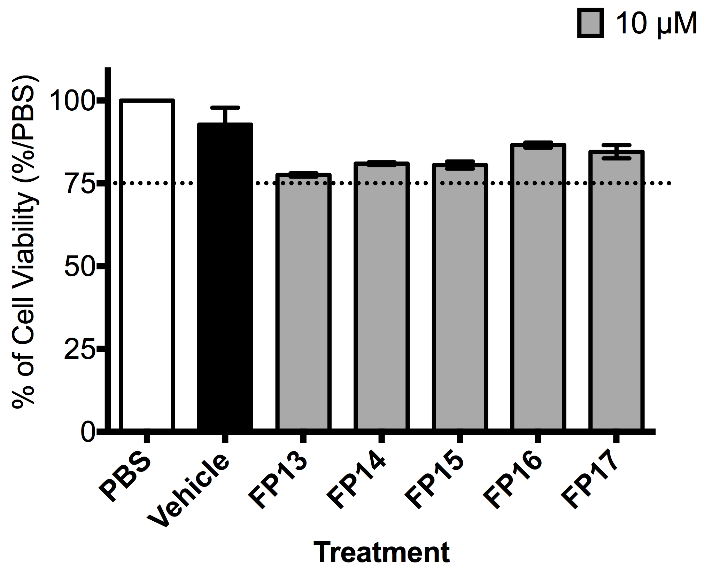
**

Figure S10**.** MTT viability assay of compounds **FP13-17** in HEK-Blue hTLR4 cells. Cells were treated with the vehicle (DMSO/EtOH 1:1) or with the indicated concentration of each compounds for 16 hours. Data were normalized with PBS administration and represent the mean of percentage ± SEM of three independent experiments.


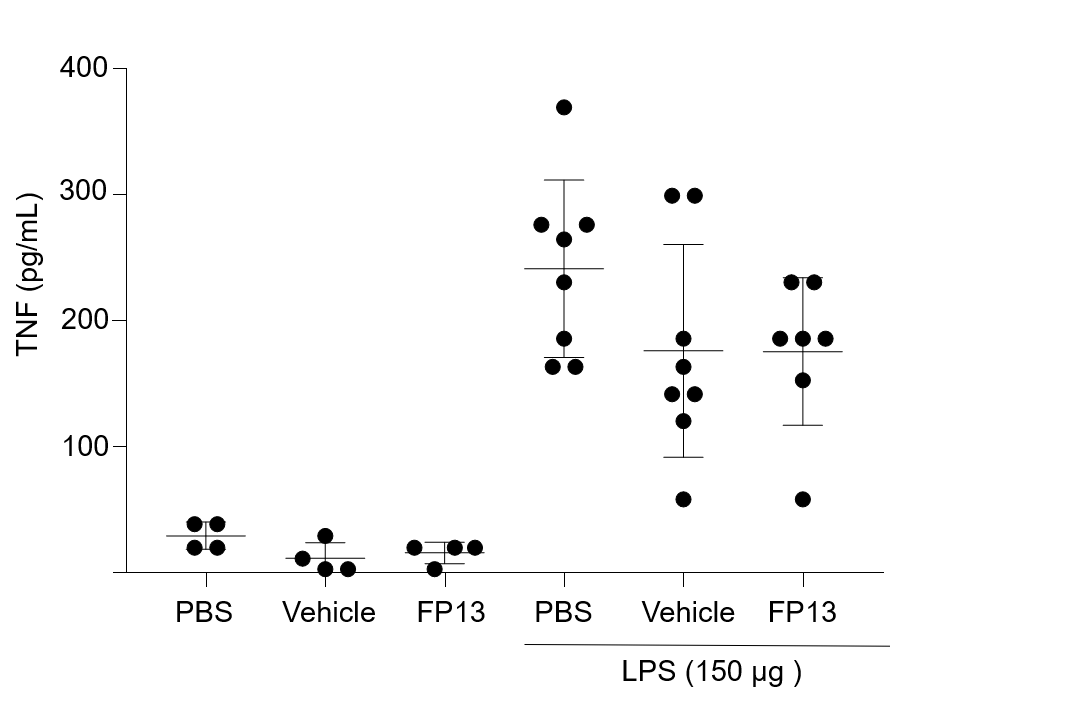


Figure S11**:** Monosaccharide **FP13** does not prevent increased serum TNF levels LPS-injected mice. C57BL/6 mice were injected i.p. with 200 µg **FP13**. 30 min later, the mice were i.p. injected with 150 µg of *Salmonella enterica* LPS, or vehicle and PBS as controls. Mice were sacrificed 4 h later and serum was collected. Serum TNF concentration was determined by Luminex-based Bio-Plex Multiplex system. Error bars represent the standard deviation of the mean.


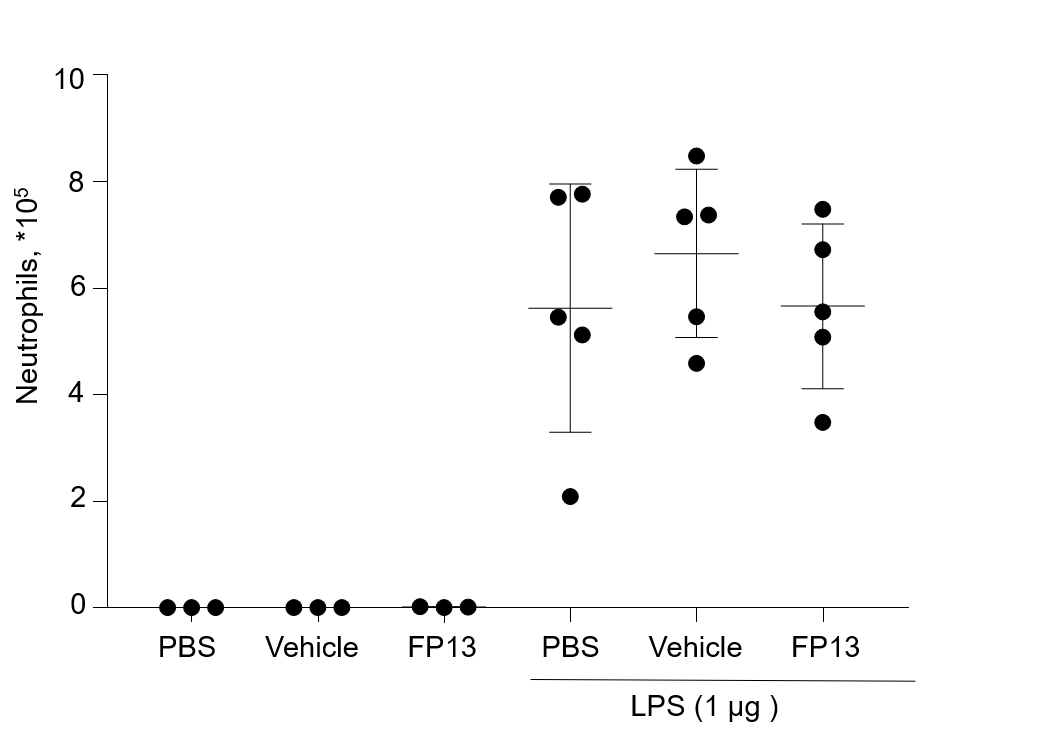


Figure S12**:** Monosaccharide **FP13** does not prevent lung neutrophilia upon administration of LPS in the lungs of mice.C57BL/6 mice were treated i.t. with 1 µg *Salmonella* LPS in the presence or absence of 45 µg **FP13**. PBS and vehicle were administered as controls. Mice were sacrificed 16 h later and neutrophilia in the BAL fluid was measured by flow cytometry. Error bars represent the standard deviation of the mean.

1. Schrödinger Release 2017-4: Maestro, Schrödinger, LLC, New York, NY, 2017. [↑](#footnote-ref-2)
